# Microglia protect against brain injury and their selective elimination dysregulates neuronal network activity after stroke

**DOI:** 10.1038/ncomms11499

**Published:** 2016-05-03

**Authors:** Gergely Szalay, Bernadett Martinecz, Nikolett Lénárt, Zsuzsanna Környei, Barbara Orsolits, Linda Judák, Eszter Császár, Rebeka Fekete, Brian L. West, Gergely Katona, Balázs Rózsa, Ádám Dénes

**Affiliations:** 1 Two-Photon Imaging Center, Institute of Experimental Medicine, Hungarian Academy of Sciences, Szigony U. 43, Budapest 1083, Hungary; 2 Laboratory of Neuroimmunology, Institute of Experimental Medicine, Hungarian Academy of Sciences, Szigony U. 43, Budapest 1083, Hungary; 3 MTA-PPKE ITK-NAP B - Two-photon measurement Technology Research Group, Pázmány Péter University, Budapest 1083, Hungary; 4 Plexxikon, Inc., Berkeley, California 94710, USA

## Abstract

Microglia are the main immune cells of the brain and contribute to common brain diseases. However, it is unclear how microglia influence neuronal activity and survival in the injured brain *in vivo*. Here we develop a precisely controlled model of brain injury induced by cerebral ischaemia combined with fast *in vivo* two-photon calcium imaging and selective microglial manipulation. We show that selective elimination of microglia leads to a striking, 60% increase in infarct size, which is reversed by microglial repopulation. Microglia-mediated protection includes reduction of excitotoxic injury, since an absence of microglia leads to dysregulated neuronal calcium responses, calcium overload and increased neuronal death. Furthermore, the incidence of spreading depolarization (SD) is markedly reduced in the absence of microglia. Thus, microglia are involved in changes in neuronal network activity and SD after brain injury *in vivo* that could have important implications for common brain diseases.

## Abstract (web summary)

How microglia contribute to brain injury or repair is unclear. Here combining microglia manipulations and calcium imaging, the authors show that selective elimination of microglia leads to disrupted neuronal calcium dynamics and markedly increased brain injury after cerebral ischemia.

## Graphical abstract

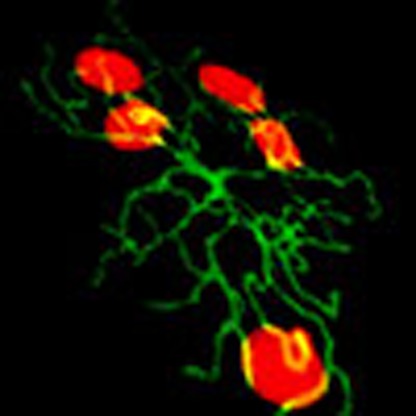

Microglia are the main resident immune-competent cell population of the central nervous system (CNS), and play an essential role in CNS development, maintenance and repair^1^. However, altered microglial activity is associated with common human diseases, such as migraine, stroke, dementia, traumatic injury, epilepsy and Parkinson's disease: these affect millions of people worldwide, representing a high socioeconomic burden^2^,^3^,^4^. In fact, microglia are capable of producing a diverse array of inflammatory mediators in response to injury or infection, and inflammation is linked with poor clinical outcome in CNS diseases^3^,^4^,^5^. At the same time, data also indicate that specific microglial actions can be neuroprotective^6^. Thus, the role of microglia in brain injury is controversial and disease dependent, whereas the mechanisms through which microglia contribute to brain injury or repair are unclear.

Microglia are highly dynamic, and constantly survey the brain parenchyma, showing rapid activation in response to harmful stimuli^7^. *In vivo* two-photon imaging studies have revealed that microglial processes interact with capillaries, react to vascular or parenchymal injury in the brain, monitor the state of synapses and remove injured neurons, their processes or synaptic structures in different models of brain injury^7^,^8^,^9^,^10^,^11^. Recently, microglia have also been shown to react to changes in extracellular calcium levels^12^. It is currently debated whether microglia could promote excitotoxicity (a major cause of neuronal death induced by calcium overload) via production of proinflammatory mediators^13^,^14^, or whether specific microglial actions could reduce excitotoxic neuronal death^15^,^16^. It is also unclear whether microglia could play a role in spreading depolarization (SD), one of the most fundamental processes of brain pathology^17^ that involves swelling of neurons, injury to dendritic spines and subsequent silencing of brain electrical activity, which is linked with excitotoxicity and general outcome in common brain diseases such as stroke, brain haemorrhage, epilepsy and migraine^18^,^19^,^20^. However, the functional role of microglia in shaping the activity of complex neuronal networks is difficult to study *in vivo*, since the large similarity between microglia and other tissue macrophages has not allowed selective *in vivo* manipulation of microglia until very recently^6^,^21^. Also, it has proved to be difficult to perform long-term monitoring of fast neuronal responses in the context of microglial activity in real time.

Microglia arise from yolk sac-derived precursors that populate the brain during early development^21^. Microglia genesis is dependent on the transcription factors IRF8 and PU.1 (ref. 22), but, once migrated to the neuroectoderm, microglia become self-renewing, which requires colony-stimulating factor 1 receptor (CSF1R)-dependent signalling^23^. High-dose treatment with a brain-penetrant CSF1R kinase inhibitor reversibly depletes microglia, an effect that has no apparent gross deleterious results in healthy mice, but that provides a powerful tool for inferring the various roles of microglia in more detail^24^.

Strikingly, we found that selective elimination of microglia from the brain leads to markedly augmented neuronal death after acute brain injury. To study the mechanisms involved, we combined fast multicolour two-photon imaging of genetically encoded calcium indicators (GECI) with a recently developed remote filament model of middle cerebral artery occlusion (MCAo) allowing us to monitor microglial responses and fast neuronal activity changes simultaneously, in real time. We show that an absence of microglia results in dysregulated neuronal responses, lack of SD and increased excitotoxic injury. Thus, supporting beneficial microglial–neuronal interactions could have profound therapeutic implications.

## Results

### CSF1R blockade leads to selective microglia depletion

**Figure 1:**
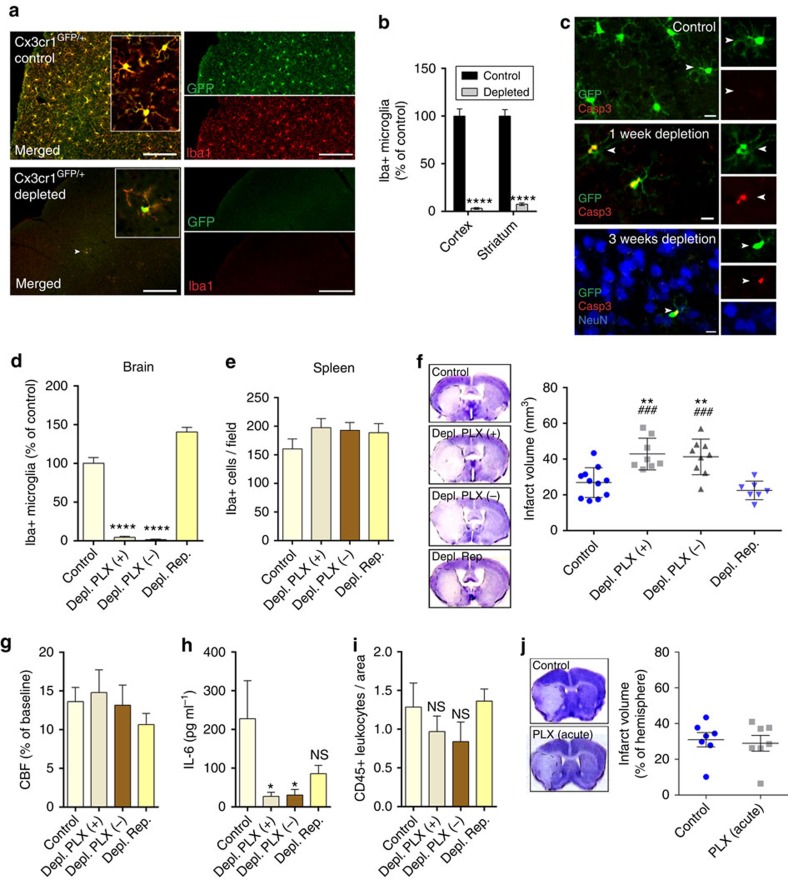
Absence of microglia results in markedly increased brain injury after cerebral ischaemia. (**a**) *Cx3Cr1*^*GFP/+*^ mice were fed a chow diet containing the CSF1R antagonist PLX3397 (290 p.p.m.) for 21 days, which resulted in an almost complete elimination of resident brain microglia. Absence of microglia was confirmed by the lack of both GFP (green) and Iba1 (red) signal. Arrowhead indicates microglia (bottom panel). (**b**) Quantification of microglia in C57BL6/J mice after control or PLX3397 diet. (**c**) PLX3397 results in microglial apoptosis as indicated by the activation of Cleaved Caspase-3 (Casp3, red) in microglia (GFP, green) *in Cx3Cr1*^*GFP/+*^ mice (arrowheads). No Caspase-3 expression is seen in neurons (NeuN, blue). (**d**) Elimination of microglia (Iba1-positive cells) from the brain is seen after feeding mice a chow diet containing PLX3397 for 21 days (Depl. PLX +). Microglia remain depleted when the diet is withdrawn for 24 h after 21 days of treatment (Depl. PLX−). Microglial repopulation occurs 2 weeks after diet withdrawal (Depl. Rep.). (**e**) No changes are seen in Iba1-positive spleen macrophages in response PLX3397 or after microglial repopulation. (**f**) An absence of microglia leads to markedly increased infarct size 24 h after cerebral ischaemia, which is reversed by microglial repopulation. (**g**) Cerebral blood flow (CBF) was measured by a laser Doppler during MCA occlusion (expressed as % of baseline). (**h**) IL-6 levels in the CSF 24 h after cerebral ischaemia. (**i**) Elimination of microglia did not increase the recruitment of CD45-positive leukocytes into the brain 24 h after cerebral ischemia. (**j**) Intraperitoneal administration of PLX3397 1 h after the onset of ischaemia did not alter brain injury as assessed 24 h after MCAo. Data are expressed as mean±s.e.m. **b**: two-way analysis of variance (ANOVA) followed by Sidak's multiple comparison, *N*=8–10; **d–i**: one-way ANOVA followed by Tukey's multiple comparison, *N*=7–11; (**j**): unpaired *t* test, *N*=7, *P<0.05, **P<0.01, ****P<0.0001 versus the control group; ^###^*P*<0.001 versus Depl. Rep; NS, not significant. Scale bars, **a**, 100 μm; **c**, 10 μm.

Microglia have been implicated in both vascular^9^,^25^ and neuronal injury^26^,^27^, and neuroprotection^28^,^29^, but tools to selectively manipulate microglia have become available only very recently^24^,^30^,^31^. To reduce the potential side effects of microglia manipulation to the minimum, we made use of the discovery that microglial survival is critically dependent on CSF1R signalling, in contrast to other tissue macrophages^23^,^24^,^31^. Feeding a chow diet containing the CSF1R antagonist PLX3397 (290 p.p.m.) for 3 weeks resulted in an almost complete elimination of microglia from the brain in *Cx3Cr1*^*GFP/+*^mice, as confirmed by the absence of green fluorescent protein (GFP)-positive cells that co-expressed the microglia/macrophage marker Iba1 (Fig. 1a). Quantitative analysis showed that after 3 weeks, 97% of microglia were eliminated from the cerebral cortex and 93% from the striatum, compared with control mice (Fig. 1b). Microglia were eliminated as a result of apoptosis, as indicated by the expression of the apoptotic marker Cleaved Caspase-3 in microglia, but not in neurons (Fig. 1c) or other cells (not shown). Correspondingly, no sign of brain inflammation, leukocyte recruitment, weight loss or increases in circulating inflammatory citokines were observed over 3 weeks of PLX3397 treatment (Supplementary Fig. 1), confirming previous results^24^,^32^. No sign of blood brain barrier (BBB) injury, as assessed by IgG penetration into the brain parenchyma was found similarly to earlier observations^24^. The number of neurons, pericytes, astrocytes and levels of the tight junction protein claudin 5 were not altered in the brain (Supplementary Fig. 2a, b) and no changes were seen in spleen macrophages and splenic or blood leukocyte populations (Supplementary Figs 3 and 4). Thus, we argued that this model of selective microglial ablation introduces minimal disturbance to the brain and does not alter peripheral leukocytes, enabling us to study the role of microglia in brain injury.

### Absence of microglia results in increased brain injury

Next, we designed a series of experiments to assess the role of microglia in brain injury and to control for the potential confounding effects PLX3397 might exert on neuronal injury. As earlier, an almost complete elimination of microglia (Iba1+ cells) from the brain was observed after feeding mice a chow diet containing PLX3397 for 21 days (Fig. 1d). Since PLX3397 is quickly cleared from central and peripheral tissues^24^, the PLX3397 diet was withdrawn for 24 h after 21 days of microglia depletion in a separate group of animals, after which microglia remained fully depleted, but only trace amounts of the drug were detected in the brain^24^. Indeed, we found that 98% of microglia were absent from the cerebral cortex 24 h after drug withdrawal (Fig. 1d). In a further group of animals, the drug was withdrawn for 2 weeks after 21 days of treatment, which led to full repopulation of microglia (Fig. 1d). No changes in splenic Iba1+ macrophages were seen in any of the experimental groups (Fig. 1e). The absence of microglia resulted in a striking, 60% increase in infarct size 24 h after MCAo (26.8 mm^3^ in control versus 42.8 mm^3^ in microglia-depleted animals), which was not affected by the presence of PLX3397, as increased infarct size (41.2 mm^3^) was also seen if PLX3397 was withdrawn 24 h before cerebral ischaemia (Fig. 1f). In contrast, repopulation of microglia completely reversed the effect of microglia depletion on increased infarct size (22.5 mm^3^), implying that the presence of microglia influences the severity of brain injury after cerebral ischaemia. We also investigated whether any of the treatment protocols affected cerebral blood flow (CBF), but no differences in CBF values were found after occlusion of the MCA (Fig. 1g) or at reperfusion (not shown) compared with baseline. Levels of interleukin 6 (IL-6)—a cytokine produced by microglia^1^—in the cerebrospinal fluid mirrored the differences seen in microglial numbers across the different experimental groups 24 h after cerebral ischaemia (Fig. 1h). Absence of microglia did not lead to increased leukocyte recruitment to the brain 24 h (Fig. 1i) and 72 h (Supplementary Fig. 5) after cerebral ischaemia or inflammation as observed previously by others after hippocampal injury^24^. In addition, Iba1-positive microglia/macrophages were still profoundly reduced in the brain 72 h after MCAo, suggesting that an absence of microglia (and larger brain injury) does not lead to significantly increased monocyte influx compared with control mice. We also investigated whether a single, intraperitoneal injection of 20 mg kg^−1^ PLX3397, which resulted in 25,930±3,600 ng ml^−1^ PLX3397 concentrations in the plasma and 1,160±290 ng g^−1^ (2.8±0.7 μM) concentrations in the intact (non-ischaemic) brain tissue (Supplementary Fig. 6) could exert any effect on neuronal injury. This regimen yields higher PLX3397 levels than that needed to maintain depletion of microglia^24^, but does not lead to microglia depletion. Mice that received a single, acute dose of 20 mg kg^−1^ PLX3397 60 min after MCAo, when BBB injury takes place based on our two-photon imaging data (see below), showed no changes in infarct size 24 h after reperfusion compared with controls (Fig. 1j), while microglial numbers were not reduced (Supplementary Fig. 6). Thus, these data suggest that an absence of microglia leads to increased brain injury independently of PLX3397 and that PLX3397 itself has no detrimental effect on neuronal injury in this model.

### Absence of microglia facilitates excitotoxicity

**Figure 2:**
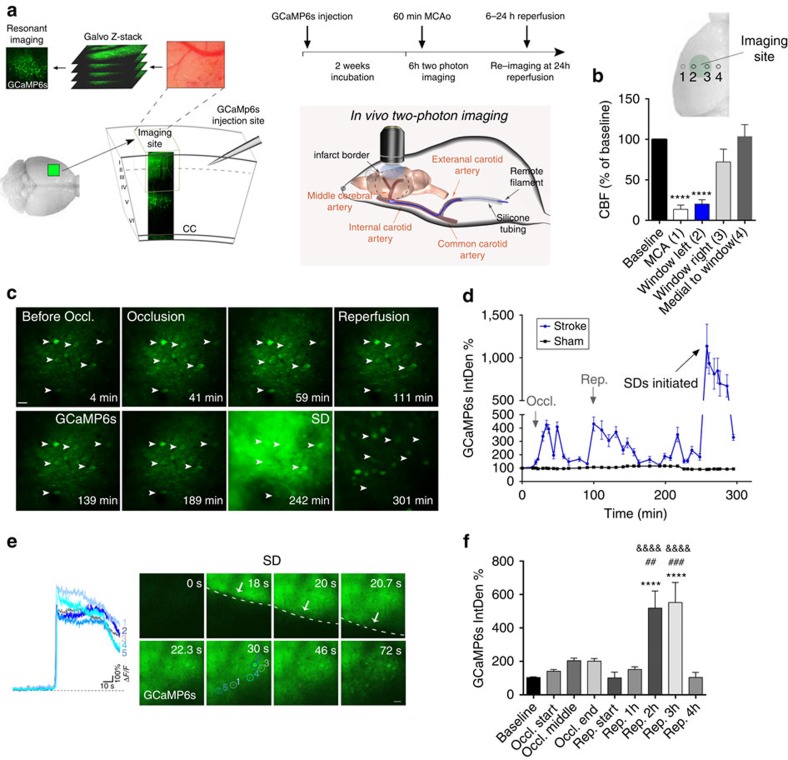
Fast *in vivo* two-photon calcium imaging reveals delayed development of excitotoxicity in the cerebral cortex after cerebral ischaemia. (**a**) AAV-mediated delivery of the genetically encoded calcium indicator GCaMP6s was performed two weeks before *in vivo* two-photon imaging with an injection site distant from the imaging area to minimize disturbance to the brain tissue. Cerebral ischaemia was induced by a remote filament approach of MCAo allowing precise control of occlusion and reperfusion during *in vivo* two-photon imaging. To monitor neuronal calcium responses in all stages of cerebral ischaemia, fast resonant scanning was performed at repeated 2 min recordings at 31.25 frames second followed by a 5-min break,continuously for up to 6 h including the assessment of baseline neuronal activity, 60 min occlusion of the MCA and 4–5 h reperfusion. (**b**) Cerebral blood flow (CBF) was measured by a laser Doppler over the MCA (1) and at different sites within (2–3) and outside (4) the area of the cranial window (*N*=5 mice). (**c**) Representative images showing GCaMP6s signal changes in a group of neurons (arrows) imaged *in vivo* before and during MCA occlusion followed by reperfusion in the cerebral cortex over a 310 min period. Images represent average intensity projections of a 2 min resonant scanning session for each data point. (**d**) Representative graph showing GCaMP6s signal changes over time in mice (*N*=4) that had been subjected to 60 min MCAo followed by 4 h reperfusion (blue line) and in sham animals (black line). Integrated density values were expressed as a percentage of baseline (IntDen%). (**e**) Spreading depolarizations (SD) are initiated 2.5–4 h after the onset of ischemia. Images show neuronal calcium changes during a single 2 min resonant scanning session, arrows indicate the wavefront of depolarization. Calcium curves from five representative neurons are shown in different colours. (**f**) Quantification of average GCaMP6s signal changes reveals delayed development of excitotoxic responses after stroke (*N*=38 neurons from 4 mice). Data are expressed as mean±s.e.m. **b**,**f**: one-way analysis of variance followed by Tukey's multiple comparison. Scale bars, **c**,**e**: 50 μm.

Next, we used *in vivo* two-photon calcium imaging combined with a recently developed remote filament model of MCAo to investigate the mechanisms through which microglia contribute to brain protection after acute injury. To this end, adeno-associated virus (AAV)-mediated delivery of the GECIs, GCaMP6s or RCaMP1 was performed using a micropipette distant from the imaging site. This minimized disturbance to the brain tissue, as evidenced by the absence of microglial activation in the imaging area where the dura mater was not injured (see Methods section and Supplementary Fig. 7a). This system enabled precise and continous monitoring of neuronal calcium changes and network activity in the cerebral cortex in real time, up to several hours post reperfusion (Fig. 2a). In a separate set of experiments, series of CBF measurements were performed by laser Doppler flowmetry to establish the site of our imaging window (30 measurements performed in *n*=5 mice). To capture neuronal GCaMP6s signal changes in the evolving infarct, the imaging site was positioned between an area with close to maximal CBF reduction (80% reduction to baseline) and an area with sub-ischaemic levels of blood flow (30% reduction to baseline, Fig. 2b). In response to induction of cerebral ischaemia for 60 min, we noticed minor increases of the GCaMP6s signal in cortical neurons and the neuropil followed by low levels of GCaMP6s signal up to 1–1.5 h after the induction of reperfusion (Fig. 2c,d). In contrast, a marked increase in intracellular calcium was observed 1.5–3 h after reperfusion in the same neurons and the neuropil caused by the initiation of SD, a basic phenomenon of brain pathology thought to contribute to excitotoxicity and linked with neuronal injury in diverse neurological diseases^18^,^20^ (Fig. 2d,e and Supplementary Video 1). Series of two to four SDs in individual mice were followed by a reduction in neuronal calcium signals by 4 h of reperfusion (Fig. 2d–f), which remained similar 24 h after cerebral ischaemia (Supplementary Fig. 7B). Quantitative analysis of GCaMP6s signal changes over time showed that significant increases in neuronal calcium levels develop only 1.5–3 h after the induction of reperfusion due to repeated SDs (Fig. 2f). The GCaMP6s signal remained unaltered in sham animals over the whole imaging period.

### Microglia contact neurons in an activity-dependent manner

**Figure 3:**
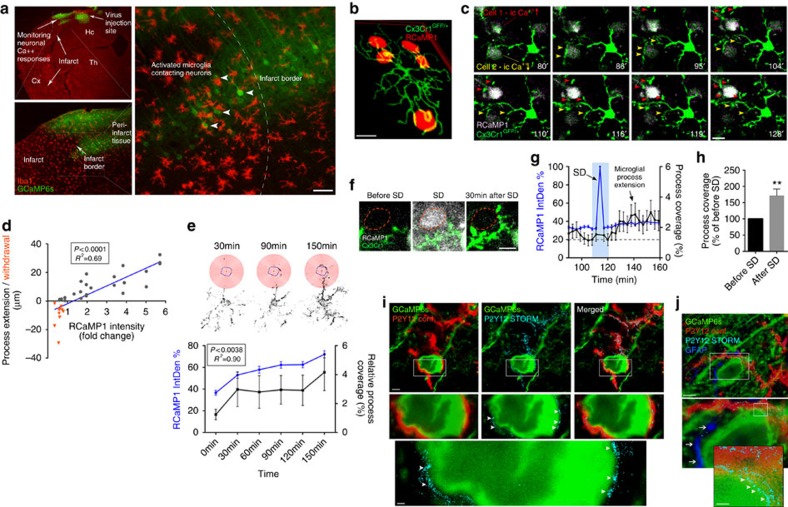
Microglia interact with neurons in an activity-dependent manner and respond to SD after cerebral ischaemia. (**a**) Activated microglial cells (Iba1, red) surround neurons showing high GCaMP6s signal (green, arrowheads), in the boundary zone of the infarct after cerebral ischaemia. (**b**) Reconstruction of *in vivo* two-photon images at different Z-planes show microglial process coverage of neurons (RCaMP1, red) in the cerebral cortex in *Cx3Cr1*^*GFP/+*^ microglia reporter mice (arrowheads) 2 h after the onset of ischaemia (**c**) *In vivo* two-photon imaging reveals microglial process extension (red arrowheads) in *Cx3Cr1*^*GFP/+*^ mice to neurons with increasing intracellular calcium levels (RCaMP1, pseudocolored) after cerebral ischaemia, whereas decreasing neuronal RCaMP1 signal is associated with microglial process withdrawal (yellow arrowheads). (**d**) Microglial process recruitment correlates significantly with changes in neuronal calcium levels after cerebral ischaemia (linear regression, *N*=35 microglial processes). (**e**) Measurement of microglial process density in concentric circles around individual neurons (modified Sholl analysis, *N*=35) showing microglial process recruitment to the neuronal cell body (circled in blue) over time after MCAo. Graph showing significant correlation between intracellular calcium levels in neurons (*N*=45) based on RCaMP1 signal changes (blue line) and microglial process coverage (black line) over time. (**f**) Spreading depolarization (SD) initiated after cerebral ischaemia rapidly increase microglial process coverage of neurons in the cerebral cortex. (**g**) Graph showing the kinetics of microglial process recruitment after SD. After SD, microglial process coverage of neurons (*N*=41) increases, even if intracellular calcium returns to near-baseline levels ((**h**), *N*=17 neurons, unpaired *t* test). Integrated density values on **e**,**g** were expressed as a percentage of baseline (IntDen%). (**i**) STORM super-resolution microscopy reveals a close contact between P2Y12-postitive microglial processes (cyan) and GCaMP6s-positive neurons (green). P2Y12 receptors form clusters at sites of microglia-neuron interaction (arrowheads). (**j**) Microglial processes and P2Y12 clusters (arrowheads) are found in close proximity to the neuronal cell membrane distant from GFAP-positive (dark blue) astrocyte processes (arrows). (**i**,**j**) Representative images from the cerebral cortex of mice subjected to MCAo. Data are expressed as mean±s.e.m. Scale bars, **a**: 50 μm; **b,c,f**: 10 μm; **i**,**j**: 5 μm (inserts 500 nm).

Several excellent previous studies have investigated microglial responses after brain injury using two-photon microscopy^1^,^7^,^8^,^10^, but *in vivo* two-photon imaging to evaluate the relationship between microglial actions and neuronal network activity has not been performed in real time. Our histological data suggested that after cerebral ischaemia, microglia and their processes surround neurons showing high GCaMP6s signal in the boundary zones of the infarct (Fig. 3a). To investigate this in real time, neuronal calcium responses were monitored with two-photon microscopy using the GECI RCaMP1, in *Cx3Cr1*^*GFP/+*^ (microglia reporter) mice (Fig. 3b). Cerebral ischaemia resulted in similar RCaMP1 changes as seen earlier with GCaMP6s. Morphological signs of microglial activation (reduced ramification and thickened processes) were seen between 1 and 4 h after the onset of ischaemia in the evolving infarct as also confirmed by Sholl analysis^33^. Microglial activation was associated with enlargement of the cell body and increased Iba1 expression from 6 h after cerebral ischaemia (Supplementary Fig. 8). Importantly, microglial processes, which were frequently found to touch neuronal cell bodies and dendrites already before MCAo, were extended to neurons showing increasing RCaMP1 signal over time, surrounding the cell body and the main dendrites, whereas microglial processes were withdrawn after prolonged reduction in neuronal intracellular calcium levels (Fig. 3b–d and Supplementary Video 2). We also assessed changes in the density of microglial processes in concentric circles around individual neurons (a modification of Sholl analysis^33^, see Methods section), which showed increasing microglial process coverage (*R*^2^=0.9, *P*=0.0038) of neurons showing a sustained rise in intracellular calcium after cerebral ischaemia (Fig. 3e). Surprisingly, we also found that SDs that typically occurred 1.5–3 h after the onset of cerebral ischaemia increased microglial process coverage of neuronal cell bodies within 30 min in both neurons showing increasing RCaMP1 signal before and after an SD (termed as Group 1 cells) and in those cells where intracellular calcium returned to near-baseline levels after SD (termed as Group 2 cells; Fig. 3f–h, Supplementary Fig. 9 and Supplementary Video 3). Super-resolution microscopy revealed that fine microglial processes form tight contacts with the neuronal cell body in the evolving infarct, whereas fewer microglia–neuron contacts were seen around the neuronal cell body in sham animals. Microglial P2Y12 receptors were found clustering at the microglia–neuron interface independently of location of astrocyte endfeet (Fig. 3i–j and Supplementary Fig 10). Thus, microglia react rapidly to changes in intracellular calcium in neurons and SD is associated with microglial process recruitment to neurons independently of sustained calcium changes in the injured brain.

### Lack of microglia alters neuronal activity and SD after injury

**Figure 4:**
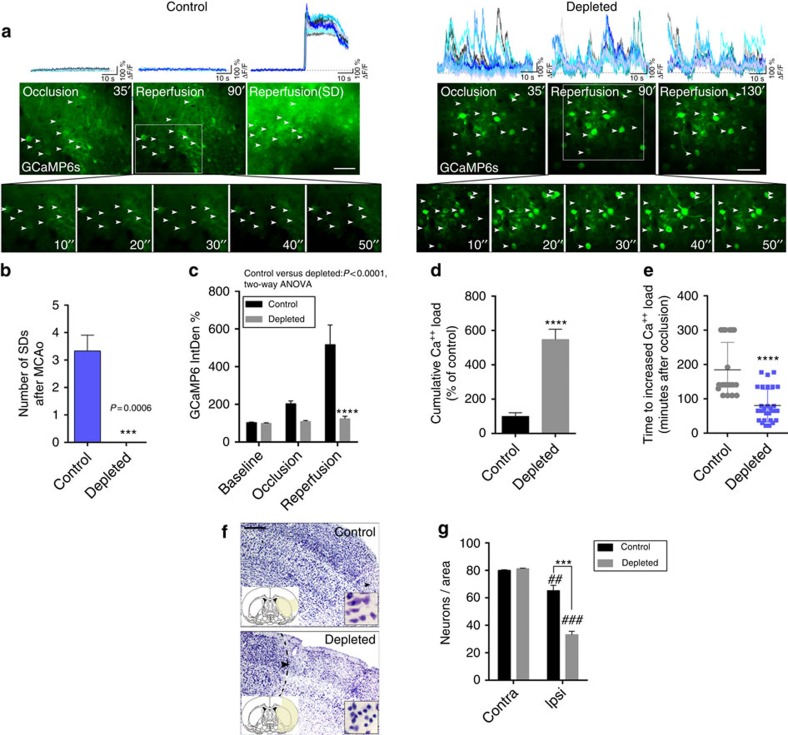
Microglia shape neuronal activity after cerebral ischaemia. (**a**) Neuronal GCaMP6s signal changes were assessed by *in vivo* two-photon imaging in the cerebral cortex using 2 min long recordings with fast resonant scanning (31.25 frames per second), separated by 5 min breaks. In control mice no significant GCaMP6s signal intensity changes were seen before the occurrence of spreading depolarizations (SD) starting typically 1.5–3 h post-reperfusion (left panel, inserts shown at Reperfusion 90′). In contrast, an absence of microglia resulted in slow neuronal oscillations (at ≈0.1 Hz frequency) already during occlusion, with similar changes seen after reperfusion (right panel). Representative calcium transients from 10–10 individual neurons (white arrows, calcium responses are indicated by different colours) are shown on the top panels from control and microglia-depleted mice. (**b**) Lack of microglia resulted in the absence of SD. (**c**) Average GCaMP6s intensity of 2 min recordings was increased at late reperfusion in control mice due to initiation of SDs, compared to microglia-depleted animals. Integrated density values were expressed as a percentage of baseline (IntDen%). (**d**) In contrast, cumulative calcium load (expressed in % of control) as calculated by summing up calcium curve integrales of individual neurons during the 2 min long two-photon recordings over a course of 5.5 h (baseline, 60 min occlusion and 4 h reperfusion) was significantly increased in microglia-depleted animals. (**e**) Microglia-depleted animals reach increased calcium load over baseline significantly earlier (due to continuous neuronal depolarizations) than control mice (due to delayed initiation of SD). (**f**) Cresyl violet staining reveals marked neuronal death in the imaging site in microglia-depleted mice compared to controls. (**g**). Quantification of cresyl violet-stained neurons (*N*=8 mice, two-way analysis of variance (ANOVA), followed by Sidak's multiple comparison) at the imaging site in the cerebral cortex and the corresponding contralateral hemisphere. Data are expressed as mean±s.e.m. **b**: *N*=4 mice per group, unpaired *t*-test; **c**: two-way ANOVA followed by Dunn's multiple comparison; **d**,**e**: unpaired *t*-test, **c**–**e**: *N*=52 neurons from 4 individual mice. ^##^*P*<0.01, ^###^*P*<0.001 versus contralateral, ***P<0.001 control versus depleted. Scale bars, **a**, 50 μm; **f**, 200 μm.

Our imaging data indicated that microglia contact neurons in an activity-dependent manner after brain injury, which led us to investigate neuronal calcium responses in the absence of microglia. To this end, two weeks after GCaMP6s delivery, repeated 2-min-long, fast two-photon imaging recordings were performed by resonant scanning, separated by 5 min breaks over a 5.5–6 h period before and after the induction of 60 min MCAo followed by reperfusion. Selective microglia depletion did not influence GCaMP6s delivery to cortical neurons (456±78 neurons per mm^2^ in control and 504±35 neurons per mm^2^ in microglia-depleted mice were GCaMP6s-positive in the imaging area before MCAo being induced). As earlier, we found that cerebral ischaemia resulted in negligible GCaMP6s signal changes in control mice during occlusion and early reperfusion, followed by the occurrence of repeated SDs 1.5–3 h after reperfusion in the evolving infarct. Calcium imaging revealed few changes in individual neurons or in the neuropil before SD (Fig. 4a, left). In contrast, in the absence of microglia striking increases in slow neuronal oscillations were observed 15–25 min after the onset of ischaemia, which continued after the induction of reperfusion up to 3–5 h post MCAo (Fig. 4a right, and Supplementary Video 4). Surprisingly, an absence of microglia was associated with the complete lack of SD in microglia-depleted animals after brain injury (Fig. 4a,b). Hence, average GCaMP6s signal levels did not increase 1.5–3 h after reperfusion as seen in control mice (Fig. 4c). However, cumulative calcium load as calculated by summing up calcium curve integrales from the 2-min resonant scanning sessions of individual neurons over an 5.5 h imaging period, was significantly (over fourfold) higher in microglia-depleted mice compared with controls (Fig. 4d). This difference was striking considering that SDs contributed substantially to cumulative calcium load in control mice, whereas no SDs were seen in the absence of microglia. Moreover, we calculated the time spent before increases in intracellular calcium load become evident in individual neurons, which was markedly shorter in the absence of microglia (Fig. 4e). To investigate whether the increased cumulative calcium load seen in the first hours after ischaemia/reperfusion is associated with subsequent neuronal injury, we investigated the fate of affected neurons at the imaging site 24 h after cerebral ischaemia. We found a significant (20%) reduction in the number of cresyl violet-stained neurons 24 h after cerebral ischaemia in control mice, which was markedly augmented (by over twofold) in microglia-depleted animals (Fig. 4f,g).

### Lack of microglia alters neuronal responses to KCl

**Figure 5:**
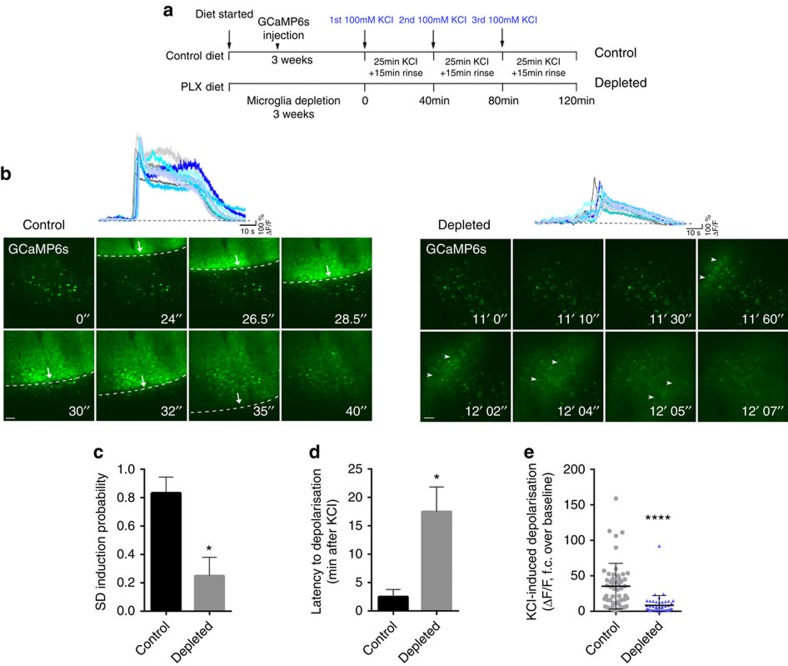
Microglia shape neuronal activity in the non-ischaemic brain (**a**) Outline of experimental procedures to investigate KCl-induced neuronal depolarization in the absence of microglia. Three weeks after feeding mice a control or a PLX3397 diet (290 p.p.m.), and two weeks following GCaMP6s delivery, spreading depolarizations (SDs) were induced by 100 mM KCl for 25 min followed by a 15 min rinse: the protocol was repeated three times. (**b**) Neurons in the cerebral cortex of control mice displayed rapid SD generation after induction by KCl (left panel, arrows indicate the evolving SD wavefront). An absence of microglia resulted in a very low incidence of SD and a low level of depolarizations compared to that seen in control animals (arrowheads indicate a pale depolarization wave seen in the absence of microglia). Representative calcium curves of 10 individual neurons are shown on the top of the panel. (**c**) SD induction probability (number of successful SD inductions/number of KCl applications) was markedly reduced in the absence of microglia. (**d**) Latency to depolarisation after KCl administration was significantly increased in microglia-depleted animals. (**e**) KCl-induced depolarization resulted in significantly lower levels of GCaMP6s signal in the absence of microglia compared to control mice. **c**,**d** and **e** graphs show mean±s.e.m. *N*=102 neurons from 4 individual mice. **c**, Mann–Whitney test; **d**,**e**, unpaired *t*-test. *P<0.05, ****P<0.0001. Scale bar, **b**, 50 μm.

To test whether an absence of microglia leads to altered neuronal calcium dynamics in the non-ischaemic brain, we applied potassium chloride (KCl) topically to the surface of the cerebral cortex, which is widely used to induce neuronal depolarization and SD^19^,^34^. Spontaneous calcium responses before KCl application and calcium responses during SD showed a good correlation with changes in local field potential (Supplementary Fig. 11). In control mice, SD was readily induced within 0.5–3 min after administration of 100 mM KCl with an obvious propagating wavefront as evidenced by GCaMP6s signal changes during fast *in vivo* two-photon imaging (Fig. 5a,b and Supplementary Video 5). In contrast, similar SD to that seen in control mice was rarely observed in the absence of microglia. In some cases a pale wavefront was seen, resulting from increases of GCaMP6s signal (considered as an SD for quantification) in neuronal cell bodies and the neuropil (Fig. 5b and Supplementary Video 6). Repeated KCl application (three times, over the course of 2 h) resulted in only 20% probability of SD in the absence of microglia as opposed to 80% in animals with functional microglia in the brain (Fig. 5c). Quantitative analysis showed that GCaMP6s signal increases in individual neurons over baseline values after KCl administration took place with significant latency (mean 17 min) in the absence of microglia, compared to control mice (2.5 min, Fig. 5d). In addition, integration of calcium curves of individual neurons revealed a marked reduction in KCl-induced depolarization in microglia-depleted mice (Fig. 5e). Thus, modulation of neuronal activity by microglia-mediated actions appears to be a basic physiological phenomenon that is evident in both the intact and the injured brain.

### Lack of microglia does not augment BBB injury after stroke

**Figure 6:**
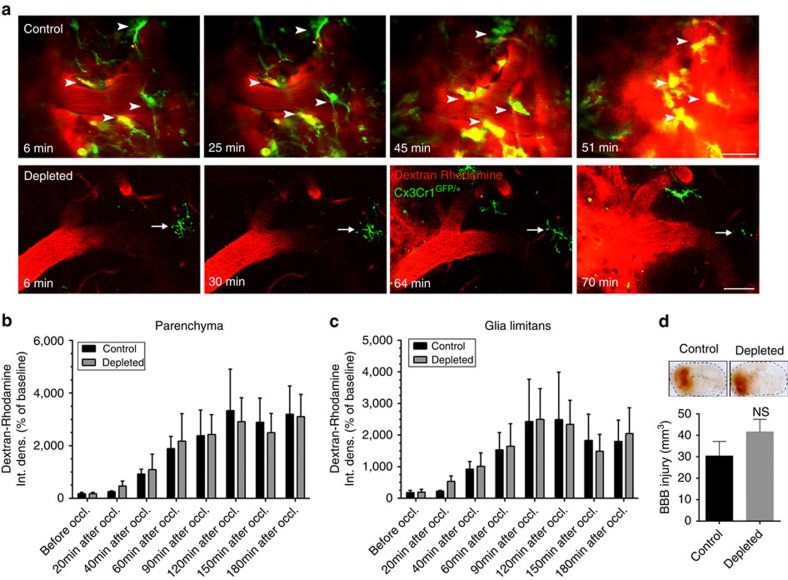
Selective depletion of microglia does not alter blood brain barrier injury after cerebral ischaemia *In vivo* two-photon imaging was performed in control mice and in mice that were fed a chow diet containing PLX3397 (290 p.p.m.) for three weeks to selectively eliminate microglia from the brain. In *Cx3Cr1*^*GFP/+*^ mice, Dextran rhodamine (1 mg per mouse in 150 μl volume) was administered via the jugular vein 15 min before MCAo. Z-stacks between 100 and 300 μm below the dura mater were recorded every 3 min over a 4–5 h period before, and after the onset of ischaemia. (**a**) *In vivo* two-photon microscopy revealed increasing Dextran Rhodamine fluorescence signal in the extravascular space indicating blood brain barrier damage following cerebral ischaemia. Areas of blood brain barrier breakdown in control mice were monitored by microglia (arrowheads), which acquire an activated phenotype over time. Remaining microglia in a microglia-depleted mouse is indicated by an arrow. (**b**) Quantification of Dextran rhodamine intensity changes in the brain parenchyma over time, before and after cerebral ischaemia in control and microglia-depleted animals. Integrated density values were expressed as a percentage of baseline. (**c**) Quantification of Dextran Rhodamine intensity changes in the glia limitans. Quantitative data (mean±s.e.m.) from *N*=12 blood vessels in control and *N*=8 blood vessels in microglia-depleted animals, from 3 mice per group, analysed with two-way analysis of variance followed by Dunn's multiple comparison. (**d**) BBB injury (mm^3^) in control and microglia-depleted mice (mean±s.e.m., *N*=8–10 mice, unpaired *t*-test) as measured by leakage of plasma-derived IgG into the brain parenchyma 24 h after MCAo. NS, not significant. Scale bar, **a**, 50 μm.

Since previous studies implicated microglia in vascular injury after cerebral ischaemia^9^, we tested whether altered BBB injury could be responsible for some of the changes we obsered in microglia-depleted animals. *In vivo* two-photon recordings of Z-stacks in *Cx3Cr1*^*GFP/+*^ mice with RCaMP1 (Fig. 3b) and histological analysis (Supplementary Fig. 12A) revealed that individual microglial cells extended processes to 6–12 nearby neurons on average, located 15–80 μm away from the microglial cell body. In addition, microglia contacting neurons were often found in the vicinity of capillaries, or extended their processes to distant microvessels. Using *Cx3Cr1*^*GFP/+*^ mice injected intravenously with a red fluorescent tracer, Dextran rhodamine (70,000 molecular weight), we set out to visualize sites of BBB injury that have been associated with altered microglial activity in previous studies^25^,^35^. BBB injury at the level of microvessels and arterioles occurred typically 50–90 min after the onset of ischaemia. Microglia located in the vicinity of blood vessels were recruited to the injured sites and displayed an activated phenotype within 15–40 min followed by partial withdrawal of their processes (Supplementary Fig. 12b). However, we found that elimination of microglia did not result in any significant alterations in the kinetics of BBB injury as assessed by *in vivo* two-photon imaging (Fig. 6) based on the extravasation of intravenously injected Dextran rhodamine in *Cx3Cr1*^*GFP/+*^ mice. Quantitative analysis showed that significant BBB injury in the brain parenchyma takes place 60 min after the onset of ischaemia (analysis of variance, *P*<0.05) in both control and microglia-depleted animals, but control and microglia-depleted animals did not significantly differ (Fig. 6b). Similarly, no differences were seen in the glia limitans in control versus microglia-depleted mice (Fig. 6c). In addition, an absence of microglia did not alter BBB injury 24 h after MCAo as assessed on coronal brain sections based on leakage of circulating IgG into the brain parenchyma (Fig. 6d). Thus, these data strongly suggest that microglia-mediated protective effects after brain injury are due to the regulation of neuronal activity by microglia and not to effects on the extent or kinetics of vascular injury. We also found blunted upregulation of IL-1α, tumour necrosis factor-α (TNFα), IL-6, monocyte chemoattractant protein-1 (MCP-1), KC (CXCL1) and IL-4 in the ipsilateral hemisphere 8 h after cerebral ischaemia in the absence of microglia (Supplementary Fig. 13), indicating that increased brain injury and changes in neuronal responses are not explained by exaggerated inflammatory responses in this model of microglia depletion.

## Discussion

Here we identify microglia as important components of the neuronal response to brain injury and show that microglia protect against neuronal injury. Selective removal of microglia from the brain leads to the dysregulation of neuronal calcium responses and network activity, increased calcium accumulation and markedly reduced SD incidence after brain injury, which is associated with profoundly increased neuronal loss. Therefore, selective targeting of the pathways involved could have a critical impact on understanding the pathophysiology of common human diseases and the development of novel therapeutic approaches.

The role of microglia in the healthy brain and in neurological diseases remains highly controversial, in spite of the substantial research effort and recent breakthroughs in this field^1^,^2^,^21^. Microglia, the resident immune cells of the brain, appear to be essential for normal brain function, whereas altered microglial function has been linked with diverse brain pathologies in both humans and experimental animals. These include diseases with a high socioeconomic burden such as depression, psychiatric diseases, epilepsy, stroke, migraine, Parkinson's disease and various forms of dementia such as Alzheimer's disease^2^,^3^,^5^,^6^,^26^,^28^,^36^. The controversies regarding the possible detrimental or protective role for microglia in different models of neurological diseases are likely due to multiple factors. Of these, the immunological and functional similarities between microglia and peripheral macrophages have made it difficult, until very recently, to selectively manipulate microglia^6^,^24^. Most microglia in the brain are protected by the BBB (except for the cells that reside in the circumventricular organs), and are hence difficult to target, while microglia become rapidly activated in response to any disturbance to the brain or after peripheral inflammatory challenges, which can shape microglial responses and outcome after different forms of brain injury^1^,^5^,^7^. It is important to note that none of the previous studies combined selective microglial depletion with high-resolution two-photon calcium imaging *in vivo*, to link microglial function with neuronal activity and injury in real time.

We, therefore, established a model of brain injury that allowed us to monitor both microglia–neuron interactions in real time and the effects of microglial depletion on neuronal activity and injury. During preliminary experiments, a model for GCaMP6s delivery was developed to minimize the disturbance of neurons or microglia at the imaging site, while the remote filament model of cerebral ischaemia allowed us to induce targeted injury in the brain without focal surgical intervention in the cerebral cortex. Elimination of microglia using CSF1R blockade was selective, as it did not deplete peripheral myeloid cells, although a small, non-significant reduction in CD11b_low_ Ly6c^vhigh^ monocytes that correspond to ∼2% of total blood leukocytes was noticed. This is unlikely to have a major impact on neuronal activity and injury in this model, especially considering the limited recruitment of monocytes/macrophages compared to microglia in the brain at 24 h reperfusion after transient focal cerebral ischaemia^37^,^38^. Depletion of microglia also had minimal impact on other cell types in the brain. This is in line with previous observations using the same approach^24^, which also demonstrated a lack of illness and behavioural alterations in microglia-depleted animals^24^,^32^. This might be due to the cessation of an essential survival signal mediated by CSF1R, leading to the apoptotic death of microglia, without the induction of inflammation. This regimen is expected to cause fewer disturbances to the brain than applying toxic substances that could lead to microglial activation or inflammation. Depletion of microglia by genetic expression of diphtheria toxin could lead to deficits in learning and contextual fear conditioning^39^, or cause cytokine expression in the brain^31^, whereas toxicity to hematopoietic cells was observed in *CD11b-HSVTK* mice in response to ganciclovir^40^. In addition, microglia ablation by CSF1R blockade did not affect astrocytes, in contrast to observations in microglia-depleted animals using the *Cx3Cr1*^*CreER*^*:iDTR* model^31^.

Excitotoxicity is considered to be a major mechanism of neuronal death that occurs due to intracellular calcium overload followed by the activation of various cell death pathways^41^. After global cerebral ischaemia, rapid, but reversible damage to dendrites and spines was found to be caused by ischaemic depolarizations immediately after cessation of blood flow^42^. SD was also observed with *in vivo* calcium imaging propagating from the core of cortical microinfarcts^43^. We observed delayed occurrence of SD in control mice after focal cerebral ischaemia with small increases in intracellular calcium during MCA occlusion. To our surprise, an absence of microglia completely prevented SD after ischaemia, and markedly reduced the incidence of SD following KCl administration in the non-ischaemic brain. SD has been associated with neuronal injury in stroke, epilepsy, migraine and other forms of brain injury^18^,^19^,^20^,^34^. *In vitro* studies on rat hippocampal brain slices have suggested that microglial polarization states could affect the SD threshold^44^. However, the lack of SD in the ischaemic brain in the absence of microglia *in vivo* was unexpected. Instead, the development of slow neuronal oscillations as early as 15–25 min after the onset of ischaemia was seen, which was not due to immediate injury to neurons, since slow oscillations were obseved even 2–3 h after reperfusion in the same cells. The majority of the affected neurons died within 24 h in the absence of microglia at the imaging site unlike in control mice, indicating that altered calcium responses could be involved in more pronounced neuronal injury as widely supported by literature data^45^,^46^. Due to the lack of research tools to influence SD independently of changing neuronal activity, no mechanistic experiments could be performed in this study to functionally link SD with neuronal injury. However, our data suggest that at least after ischaemia, neuronal death in complex neuronal networks following a series of SDs could be lower than in response to dysregulated neuronal network activity and long-lasting oscillations as seen in the absence of microglia. Altered neuronal responses were observed already 30–45 min before BBB injury in microglia-depleted animals after stroke and were also seen after KCl administration, which does not lead to the disruption of the BBB. Therefore, these data suggest that microglia could impact on neuronal calcium responses and protect against neuronal injury independently of vascular injury. Mice were anaesthetized during imaging, which could alter neuronal responses^47^. However, control and microglia-depleted mice were exposed to uniformly low isoflurane levels (between 0.8 and 1.1%), hence the anaesthetic is unlikely to contribute to the profoundly altered neuronal responses seen in the absence of microglia. We have not found differences in CBF after MCA occlusion and reperfusion between control and microglia-depleted mice either, although we are aware that laser Doppler measurements only reveal changes in blood flow relative to baseline values in the superficial areas of the cerebral cortex.

We have also investigated whether PLX3397 has any effect on neuronal injury independently of microglia depletion. It has been postulated that CSF1R signalling could contribute to neuronal survival independently of microglia^48^. However, in our experiments withdrawal of PLX3397 for 24 h, which reduces drug concentrations to below detection level^24^, while microglia remained depleted, resulted in increased brain injury, similar to that seen in mice fed a PLX3397 diet at the time of MCAo. Increases in brain injury were completely reversed after repopulation of microglia. In addition, acute treatment with a PLX3397 dose higher than that needed to block microglial repopulation^24^ did not result in depletion of microglia and did not alter brain injury. It is expected that BBB injury after cerebral ischaemia would further increase PLX3397 levels in the brain. These experiments suggest that larger brain injury in this model is due to the absence of functional microglia. Data from previous studies also support our conclusions. Elimination of microglia from rat organotypic slice cultures in a CSF1R-independent manner reduced the incidence of SD, whereas dampening of microglial M1 signalling by minocycline or conditioned medium from M2a-polarized primary microglia led to higher SD threshold^44^. In addition, inhibition of proliferating microglial cells in *CD11b-TK*^*mt-30*^ transgenic mice resulted in larger ischaemic injury^49^. The effects of microglia depletion on cerebral perfusion, somatosensory and motor responses, delayed phases of neuronal injury and functional recovery, will need to be investigated in future studies using models of brain injury and neurodegeneration.

The exact molecular mechanisms through which microglia sense and regulate neuronal activity are presently unclear but investigation of the molecular pathways involved extends beyond the scope of this already complex study. It is likely that microglia–neuron signalling involves highly complex interactions between metabolic pathways, neurotransmitters and their receptors, ion channels and inflammatory mediators. Microglia–neuron interactions in the optic tectum of the larval zebrafish were found to be dependent on neuronal pannexin-1 hemichannels and the small Rho GTPase, Rac in microglia^50^. ATP triggers microglial migration and process extension in both zebrafish larvae and the mammalian brain *in vivo*^8^,^50^. In fact, super-resolution microscopy revealed increased microglial process coverage of neuronal cell bodies after ischaemia and clustering of P2Y12 receptors on fine microglial processes at sites of microglia-neuron contacts, although the functional role of purinergic signalling was not investigated in this study. Glial fibrillary acidic protein (GFAP)-positive astrocyte processes have not been observed at the microglia–neuron interface, but it is possible that some microglial actions on neurons could be mediated via astrocytes. Microglia are known to produce a diverse array of inflammatory mediators that act directly on neurons and impact on neuronal activity and injury. Some of these, such as IL-1 potentiate excitotoxicity and neuronal injury^5^, while TNFα is known to alter the balance between neuronal excitation and inhibition via different, complementary mechanisms that involve neuronal AMPA, NMDA and GABA_A_ receptors^51^. Despite larger brain injury, an absence of microglia was associated with blunted ischaemia-induced production of both proinflammatory cytokines (IL-1α, TNFα) and cytokines that are considered to be neuroprotective in the brain (IL-6, IL-4)^52^,^53^. Thus, the possible contribution of these cytokines to brain injury remains to be investigated further. The net effect of different microglial actions on neuronal activity and injury could be highly dependent on the dominance of certain pathophysiological mechanisms in different CNS diseases. However, our data are the first to demonstrate that microglia markedly protect neurons against excitotoxicity and injury after cerebral ischaemia *in vivo*, in spite of the plethora of inflammatory mediators they produce.

Taken together, our results obtained by the combination of high speed *in vivo* two-photon imaging with selective microglia manipulation revealed a previously unrecognized link between protective microglial actions and neuronal activity and injury. These results could have profound implications on the pathophysiology and potential treatment of major human diseases.

## Methods

### Mice

Experiments were carried out in 12–16 weeks old male C57BL/6J and *Cx3Cr1*^*GFP/+*^mice, breeding in the specific-pathogen-free (SPF) unit of the Institute of Experimental Medicine. Animals were allowed free access to food and water and were maintained under controlled temperature, humidity and light conditions. All procedures were in accordance with STAIR and ARRIVE guidelines, and the guidelines set by the European Communities Council Directive (86/609 EEC) and the Hungarian Act of Animal Care and Experimentation (1998; XXVIII, section 243/1998), approved by the Animal Care and Use Committee of the Institute of Experimental Medicine.

### Selective elimination and repopulation of microglia

PLX3397 was provided by Plexxikon and formulated in AIN-76A standard chow by Research Diets (290 p.p.m.; 290 mg PLX3397 in 1 kg chow). Mice were fed a PLX3397 diet for 3 weeks to eliminate microglia^24^. One group of mice was given PLX3397 for 3 weeks, followed by withdrawal of the drug by replacing the PLX3397 diet with control chow diet for 24 h before experiments. Another group of mice was fed PLX3397 for 3 weeks followed by 2 weeks on control diet to allow for the repopulation of microglia in the brain. In agreement with earlier results^24^, we have not found any obvious behavioural alterations, weight loss or sign of illness in mice fed a PLX3397 diet for 3 weeks or longer. Individual mice were observed for a 5-min period once daily for any changes in activity, exploratory behaviour and social interaction. Baseline temperature was recorded for every mouse before surgery or imaging. No mice were excluded from these studies due to fever, weight loss, infection or behavioural alterations as a result of PLX3397 diet.

### AAV vector injection

The injection procedure was performed as described previously^54^, with some modifications. A 0.5-mm hole was opened in the skull with the tip of a dental drill over the S1 cortical region (centred 1.5 mm lateral and 1.5 mm posterior to the bregma). A glass micropipette was lowered 300 μm under the pia, at a 60° angle towards the imaging area to minimize tissue disturbance at the imaging site, followed by slow injection (300 nl over 5 min) of either AAV1.Syn.GCaMP6s.WPRE.SV40 or AAV1.Syn.RCaMP1h.WPRE.SV40 viral vectors (Penn Vectore Core, Philadelphia, PA; titre 6.32 × 10^11^ IU ml^−1^).

### Cranial window surgery

Two weeks after injection mice were anaesthetized using isoflurane (3% for induction, 1.5–2% during surgery). First, a custom-made aluminium head plate was fixed to the skull using cyanoarcylate glue and dental cement. Then, the skull was either thinned to 20 μm over the imaging site or a circular craniotomy (3 mm diameter) was made above the S1 cortex (centred 2.7 mm lateral and 1 mm posterior to the bregma) without touching the dura mater. During drilling, the place of craniotomy was rinsed continuously with cold Ringer solution. The location of the imaging window was defined in a separate set of experiments by series of CBF measurements using laser Doppler flowmetry (30 measurements performed at 6 different sites, in *n*=5 mice). This was done by recording baseline blood flow followed by the occlusion of the MCA with an intraluminal filament for ∼15 s after which reperfusion was induced and the Doppler probe repositioned repeating this procedure several times between the MCA and the midline in the same mouse. The area of the craniotomy was covered with a 3-mm cover glass and fixed with dental cement. In preliminary experiments, microglial activation and neuronal calcium responses were assessed in the case of thinned skull preparations and after craniotomy without dura mater injury. No changes in neuronal calcium signals, microglial CD45, Iba1 or *Cx3Cr1* expression were found in either case as opposed to craniotomy with durectomy (Supplementary Fig. 1, and not shown).

### Imaging fast calcium responses and BBB injury

All experiments were performed on a Femto2D-DualScanhead microscope (Femtonics Ltd., Budapest, Hungary). Laser pulses were generated by a Mai Tai HP laser (SpectraPhysics, Santa Clara, CA) or by a Chameleon Ultra II laser (Coherent, Inc., Santa Clara, CA). The wavelength was set to 910 nm for GCaMP6s and to 980 nm when RCaMP1 and microglia in *Cx3Cr1*^*GFP/+*^mice were measured simultaneously. For excitation and signal collection, a CFI75 LWD 16XW/0.8 lens (Nikon, 16 ×, NA 0.8) was used, separated using a dichroic mirror (700dcxru, Chroma Technology) before the two channel detector unit, which was sitting on the objective arm (travelling detector system) as described in detail elsewhere^55^,^56^. The DualScanhead microscope (Femtonics Ltd., Budapest, Hungary) is coupled with two parallel light paths, which allows imaging at the same imaging site either with a galvanic mirror or with resonant scanner without moving or changing the sample. First, the galvo-scanning light path was used to acquire Z-stacks from 200 to 80 μm under the dura mater, with 12 images per stack at 800 × 800 pixel resolution (0.75 μm per pixel). Then, the light path was changed to the resonant scanning mode and two-photon imaging at video rate (31.25 frames per second) was performed at 512 × 512 pixel resolution (1 μm per pixel) for 60–120 s. This protocol was repeated every 5 min over the whole (5.5–6 h) imaging period for cerebral ischaemia studies. Data acquisition was performed by MesC softver (Femtonics Ltd.). To image microglia in *Cx3Cr1*^*GFP/+*^ mice simultaneously with the assessment of BBB injury the galvo-scanning light path was used. Z-stacks were recorded in the cerebral cortex (same area as used for neuronal calcium measurements) continuously from 200 μm to 110 μm under the dura mater every 3 min for a 5–6 h period before, and after the onset of ischaemia. Integrated density (area of the ROI × the mean of the ROI) was assessed at 6–6 randomly selected areas over the brain parenchyma and the glia limitans in every stack, using ImageJ. Integrated density values were expressed as a percentage of baseline (IntDen%). The occlusion of the MCA during *in vivo* two-photon imaging was verified by blood flow velocity changes measured using the line-scan mode of the galvo-scanning light path. Dextran rhodamine signal was recorded along a linear scanning trajectory perpendicular to the blood vessels' wall (measuring a single, 1.5 μm linear line with 3–5 kHz temporal resolution) on the red channel. Blood flow velocity was calculated as earlier^57^. Three mice in total were excluded from the imaging studies *pre hoc*, one control mouse after cerebral ischaemia due to improper laser intensity during imaging and two (one control and one microglia depleted) mice from KCl studies due to inappropriate anaesthesia.

### SD induction with KCl solution

In one group of mice, a small area of the cranial window was left uncovered laterally to the cover glass (where the dura mater was not injured). The dura mater was focally opened using a syringe needle and SD was induced by applying 100 mM KCl solution to the surface of the brain for 25 min. Three repeated SD induction periods were recorded. During SD induction, animals were continuously imaged with resonant scanning for repeated 2 min periods. Then, a 15-min resting period was put in the protocol when the KCl solution was replaced with Ringer solution until the beginning of the next SD induction. In a set of studies, local field potential was measured simultaneously with GCaMP6s signal changes before and after KCl application, using a borosilicate glass electrode (∼0.1 MΩ) filled with ACSF. Recordings were made in IC mode at 0 mV holding potential.

### Remote filament model of MCAo

Cranial window preparation was followed by remote filament implantation under isoflurane anaesthesia. The common carotid, the external carotid and the internal carotid arteries were exposed and the common carotid artery temporarily clipped. A 5 cm long, 5.0 monofilament with silicon rubber coating (210 μm tip diameter), residing in a silicone guiding tube was introduced into the left external carotid artery and advanced along the internal carotid artery up to 8 mm, without occluding the MCA. Blood flow was restored in the common carotid artery and the silicone guiding tube was secured, followed by closure of the wound at the site of surgery, to allow free manipulation of the monofilament during *in vivo* two-photon imaging. Mice were positioned into the two-photon microscope, after which 15–30 min baseline imaging was performed followed by the induction of 60 min of MCA occlusion and 4–5 h reperfusion.

### Middle cerebral artery occlusion

MCAo was performed using the intraluminal filament technique as described earlier^58^. In brief, animals were anaesthetized with isoflurane and a silicone-coated monofilament (210 μm tip diameter, Doccol, US) was introduced into the left external carotid artery and advanced along the internal carotid artery to occlude the MCA for 45 min. Occlusion was confirmed by a laser Doppler (Moor Instruments, UK). During surgery, core temperature was maintained at 37±0.5 °C. After experimental stroke, four mice were excluded pre hoc due to incomplete occlusion of the MCA or surgical artifacts.

### Tissue processing and immunostaining

After terminal anaesthesia, mice were perfused transcardially with saline followed by ice-cold 4% paraformaldehyde. Brains and spleens were post-fixed for 24 h, cryoprotected in 10% sucrose/PBS and sectioned (25 μm diameter) on a sledge microtome. Detection of IgG leakage to the brain parenchyma by immunostaining (Vector, NBA-2000, horse anti-mouse biotynilated IgG 1:500) was used to assess BBB permeability on brain sections as described earlier^58^. Immunofluorescence was performed on free-floating brain sections using combinations of rabbit anti-Iba1 (WAKO, 019-19741, 1:500), rat anti-CD45 (AbDSerotec, MCA1388, 1:250), mouse anti-GFAP (Sigma, G3893, 1:500), rabbit anti-Claudin-5 (Invitrogen, 34-1600, 1:500) and goat anti-PDGFRβ (R&D Systems, AF1042, 1:500) antibodies. Sections were incubated in a primary antibody cocktail overnight followed by adequate fluorochrome (Invitrogen, A21206, donkey anti-rabbit Alexa 488 1:500; Invitrogen, A21203, donkey-anti-mouse Alexa 594 1:500)-conjugated antibodies. Biotinylated tomato lectin (Sigma-Aldrich, L0651-1 mg, 1:100) was used to visualize blood vessels, followed by streptavidin Alexa 350 conjugate (Invitrogen, S11249, 1:200). Images were captured using a Zeiss Axiovert 200M microscope with Axiovision 4.8 software.

### Super-resolution microscopy

Free-floating brain sections were blocked with 2% normal donkey serum followed by immunostaining with rabbit anti-P2Y12 (Anaspec, AS-55043A, 1:500) antibody and anti-rabbit Alexa 647 secondary antibody (Jackson ImmunoResearch, 711-605-152, 1:400). Neurons were visualized by GCaMP6s signal and astrocytes detected using a mouse anti-GFAP antibody (Sigma, G3893, 1:500) developed by anti-mouse DyLight 405-Streptavidin secondary antibody (Jackson ImmunoResearch, 016-470-084, 1:400). Sections were mounted onto #1.5 borosilicate coverslips and covered with imaging medium containing 5% glucose, 0.1 M mercaptoethylamine, 1 mg ml^−1^ glucose oxidase, and catalase (Sigma, 1500 U ml^−1^) in Dulbecco's PBS (Sigma), immediately before imaging^59^. STORM imaging was performed for P2Y12 (stimulated by a 647 nm laser) by using a Nikon N-STORM C2+ super-resolution system that combines ‘Stochastic Optical Reconstruction Microscopy' technology and Nikon's Eclipse Ti research inverted microscope to reach a lateral resolution of 20 nm and axial resolution of 50 nm (refs 60, 61).

### Cytokine measurement

IL-6 was measured in cerebrospinal fluid samples and IL-1α, IL-1β, TNFα, IFNγ, IL-6, MCP-1, RANTES (CCL5), G-CSF, KC (CXCL1), IL-10 and IL-4 were measured in homogenates of saline perfused ipsilateral and contralateral brain hemispheres^62^ by cytometric bead array using CBA Flex Sets (all from BD Biosciences, 560157, 560232, 558299, 558296, 558301, 558342, 558345, 560152, 558340, 558300, 558294, respectively). Measurements were performed on a BD FACSVerse machine and data analysed using FCAP Array software (BD Biosciences). Brain tissue cytokine levels were corrected for the total protein content of the samples as measured by BCA assay (Pierce).

### Flow cytometry

Following Fc receptor blockade (eBioscience, 16-0161-85 anti-mouse CD16/CD32, 1:100), blood cells and splenic leukocytes cells were stained with cocktails of selected antibodies: T cells (all from eBioscience, 11-0043-82 anti-mouse CD4-FITC, 1:100; 12-0081-82 anti-mouse CD8a-PE, 1:400; 17-0031-80 anti-mouse CD3e-APC, 1:200), B cells (all from eBioscience, 11-0193-81 anti-mouse CD19-FITC, 1:100; 17-5321-81 anti-mouse MHCII (I-A/I-E)-APC, 1:400); granulocytes/monocytes (all from eBioscience, 11-0112-81 anti-mouse CD11b-FITC, 1:100; 25-5932-80 anti-mouse Ly-6C-PE-Cy7, 1:400) and macrophages (BD Biosciences, 563900 rat anti-mouse F4/80-like receptor-Violet, 1:200). Antibodies were purchased from eBioscience. Cells were acquired on a BD FACSVerse flow cytometer (BD Biosciences, US) and data were analysed using FACSuite software (BD Biosciences). Total blood cell counts were calculated by using 15 μm polystyrene microbeads (Polysciences, 18328-5).

### Quantitative analysis

Animals were randomized for MCAo experiments using GraphPad Random Number Generator. All quantitative analysis was performed under blinded conditions. The volume of ischaemic brain damage was measured on cresyl violet-stained brain sections. Infarct size and BBB injury at 24 h reperfusion were calculated by integration of areas of damage measured at eight neuroanatomically defined coronal levels (between 2.9 mm rostral and −4.9 mm caudal to bregma) with the distance between coronal levels followed by correction for oedema, as described previously^58^. Quantitative analysis of immunostaining was performed on at least three, randomly selected fields within the region of interest for each brain section, on 4-4 serial coronal sections separated by 400 μm for the striatum and 800 μm for the cerebral cortex (between 0.8 posterior and 0.8 anterior and between 2.4 posterior and 0.8 anterior to the bregma, respectively). The border of infarct was defined on cresyl violet-stained brain sections and microglial activation was also visualized on adjacent sections based on Iba1 immunofluorescence. Two-photon image sequences were analysed using ImageJ. Measurement units were collected from MesC files with HDF5 importer plugin. Background intensity was measured in a dark region of the field of view and was subtracted from each image sequence. The GCaMP6s signal of individual neurons was collected with ImageJ Plot-axis Z profile into the MES (Femtonics Ltd.) curve analysis modul. The trace of the individual cells was normalized to the baseline level of signal (a time interval without calcium activity, typically the begining of the imagig sequence). Then, the area under the curve was calculated for calcium curves after cerebral ischaemia or SD induction with KCl. Microglia-neuron interactions were assessed on Z-stacks recorded every 3 min between 200 and 11 μm under the dura mater. Microglia contacting neurons were randomly selected and neurons showing at least 1.5-foldchange in RCaMP1 signal were used to study microglial process recruitment over a 150 min period after the onset of ischaemia. The net extension/withdrawal of microglial processes to individual neurons was plotted against relative RCaMP1 signal intensity changes measured during the same period. Changes in microglial ramification were investigated with Sholl analysis as described earlier^33^. Two-photon images of individual microglial cells were converted into a minimum intensity projection image using the concentric circles plugin of the ImageJ with a 2 μm step size. The intercesctions between the circles and the cell processes were calculated manually for each cell. To evaluate microglia process recruitment to neuronal soma, a concentric circle macro (modification of a Sholl analysis) was written for Fiji. Microglia process coverage around neurons was assessed in 30 circles (0.5 μm step size) with an r step of 4. For three-dimensional reconstruction of *in vivo* two-photon images at different Z-planes to visualize the relationship between neuronal RCaMP1 and microglial processes in *Cx3Cr1*^*GFP/+*^mice, the Simple Neurite Tracer plugin (Fiji) was used.

### Statistics

Group sizes for MCAo studies with infarct size assessment were determined by power calculation (GPower 3.1) based on results from our representative previous studies (α error probability: 0.05, power: 0.8 and an estimated 20% s.d.). This resulted in *n*=7 for two experimental groups (Cohen's *d* effect size: 1.66) and *n*=9 for four experimental groups (effect size f: 0.6). No formal power calculation was performed for imaging studies. Data were analysed using Student's *t*-test or Mann–Whitney test (comparing two experimental groups), one-way or two-way analysis of variance followed by Tukey's and Sidak's *post hoc* comparison, respectively (comparing three or more groups) and linear regression was used for correlation analysis (GraphPad Prism 6.0). *P*<0.05 was considered statistically significant.

## Additional information

**How to cite this article:** Szalay, G. *et al*. Microglia protect against brain injury and their selective elimination dysregulates neuronal network activity after stroke. *Nat. Commun.* 7:11499 doi: 10.1038/ncomms11499 (2016).

## Supplementary Material

Supplementary Information Supplementary Figures 1-13

Supplementary Movie 1 Spreading depolarization induced after cerebral ischemia in the cerebral cortex. In vivo two-photon imaging of GCaMP6s signal changes showing induction of spreading depolarization in the cerebral cortex after cerebral ischemia (see Figure 2E for details). Imaging was performed with resonant scanning at 31.25 frames / s.

Supplementary Movie 2 Microglia react rapidly to changes in neuronal intracellular calcium levels after cerebral ischemia. Increases in neuronal RCaMP1 signal (white to-magenta, pseudocolored) are associated with microglial process extension (green) after cerebral ischemia in Cx3Cr1GFP/+ (microglia reporter) mice (see Figure 3C for details). In vivo two-photon images were recorded with Galvo scanning every 3 min.

Supplementary Movie 3 Microglial process recruitment to neuronal cell bodies increases rapidly after spreading depolarization. Neuronal RCaMP1 signal (white, pseudocolored) showing spreading depolarization (SD), which is followed by microglial process recruitment (green) after cerebral ischemia in Cx3Cr1GFP/+ (microglia reporter) mice (see Figure 3F for details). In vivo two-photon images were recorded with Galvo scanning every 3 min.

Supplementary Movie 4 Absence of microglia leads to slow neuronal oscillations and lack of spreading depolarization after cerebral ischemia in the cerebral cortex. In vivo two-photon imaging of GCaMP6s signal changes showing the development of slow neuronal oscillations, which is associated with lack of spreading depolarization in the cerebral cortex after cerebral ischemia in microglia-depleted animals (see Figure 4A for details). Imaging was performed with resonant scanning at 31.25 frames /s.

Supplementary Movie 5 Spreading depolarization induced by KCl in the cerebral cortex. In vivo two-photon imaging of GCaMP6s signal changes showing induction of spreading depolarization in the cerebral cortex after administration of 100 mM KCl (see Figure 5A for details). Imaging was performed with resonant scanning at 31.25 frames /s.

Supplementary Movie 6 Absence of microglia reduces spreading depolarization after KCl administration in the cerebral cortex. In vivo two-photon imaging of GCaMP6s signal changes reveals inhibition of spreading depolarization in the cerebral cortex after administration of 100 mM KCl (see Figure 5A for details) in microgliadepleted animals. Imaging was performed with resonant scanning at 31.25 frames /s.
