# Supplementary material for: Microglia protect against brain injury and their selective elimination dysregulates neuronal network activity after stroke
Source: Nat Commun. 2016 May 3;7:11499. doi: 10.1038/ncomms11499 (PMC4857403; doi:10.1038/ncomms11499)
Supplement: Supplementary Information — Supplementary Figures 1-13 [file ncomms11499-s1.pdf]

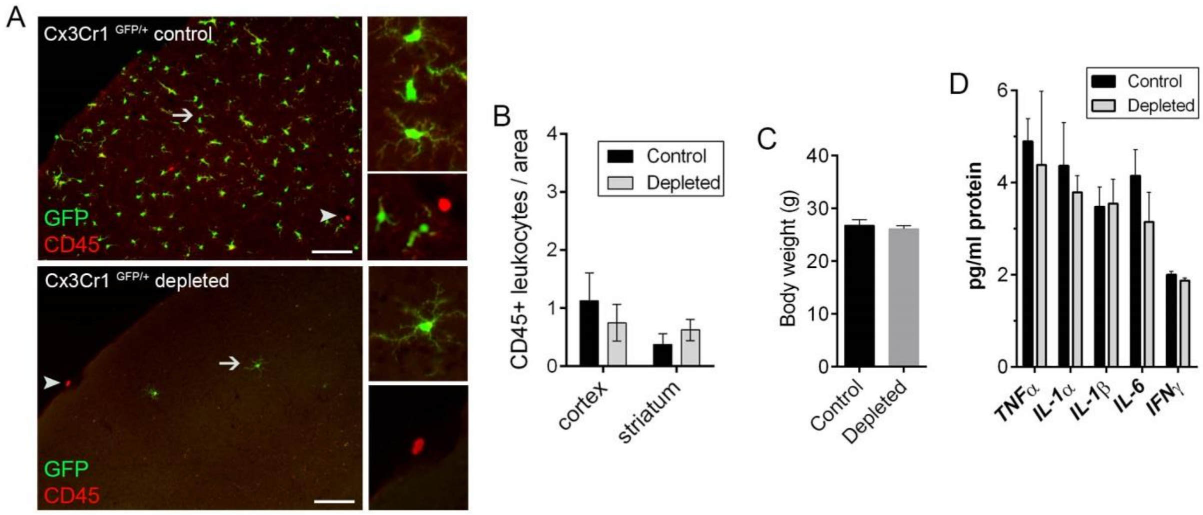

Supplementary Figure 1. **Selective depletion of microglia does not result in leukocyte infiltration into the brain and does not cause illness or weight loss. A.** In Cx3Cr1$^{GFP/+}$ (microglia reporter) mice an almost complete elimination of microglia (arrows, green) is seen after feeding mice a chow diet containing PLX3397 (290 ppm) for 21 days. Depletion of microglia does not result in recruitment of CD45+ leukocytes (arrowhead, red) into the brain. **B**. No infiltration of CD45+ leukocytes is seen in the brain parenchyma in C57BL6/J mice as quantified in the striatum and the cerebral cortex. **C**. Microglia depletion does not result in weight loss in mice fed a chow diet containing PLX3397 (290 ppm) for 21 days. **D**. Plasma cytokines were measured by cytometric bead array after feeding mice a control or a PLX3397 diet for 21 days. Data are expressed as mean ± s.e.m., n=8-10. B, D: two-way ANOVA followed by Dunn's multiple comparison; C: unpaired t test. Scale bar: A, 50 µm.

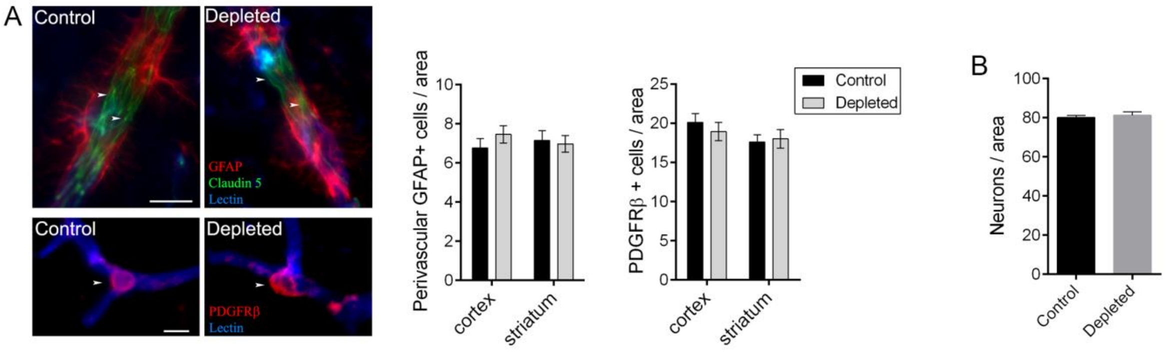

Supplementary Figure 2. Selective elimination of microglia does not affect other cell types in the brain. A. Depletion of microglia does not result in any changes in the morphology or the number of perivascular astrocytes (GFAP, red, top panel), or endothelial cells (lectin, blue) expressing the tight junction protein claudin-5 (green, arrowheads). Depletion of microglia does not result in any changes in the morphology or the number of pericytes (PDGFRβ+ cells, red, bottom panel). **B**. Depletion of microglia does not result in any changes in the number of cresyl violet–stained neurons in the cerebral cortex. Data are expressed as mean ± s.e.m., n=8. A: two-way ANOVA followed by Dunn's multiple comparison; B: unpaired t test. Scale bars: A, top panel 20 μm, bottom panel 10 μm.

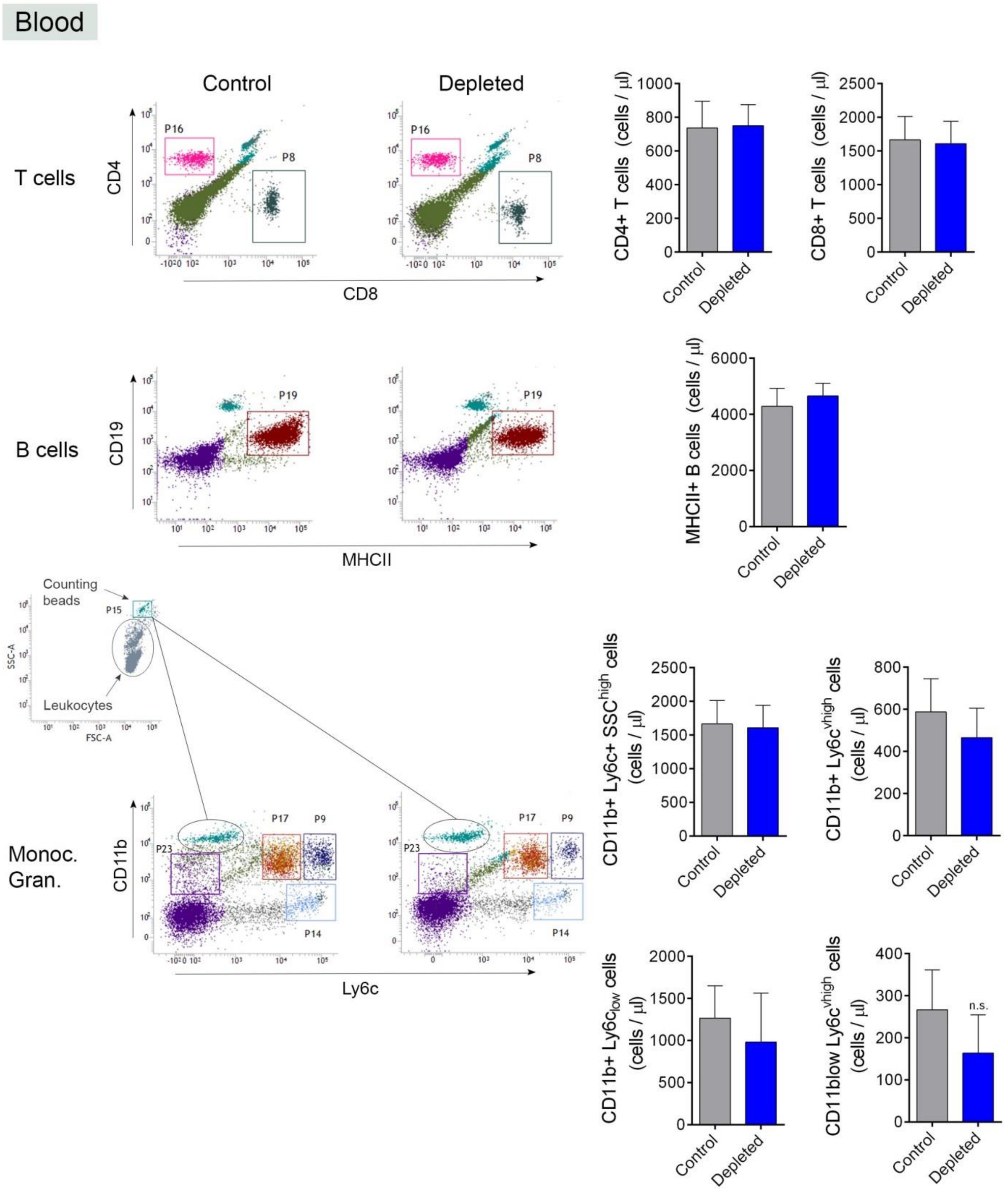

Supplementary Figure 3. **Selective depletion of microglia does not alter main blood cell populations.** C57BL6/J mice were fed a chow diet for 21 days containing the CSF1R antagonist PLX3397 (290 ppm). Blood samples were labelled with mixtures of specific antibodies against leukocyte populations and subjected to flow cytometric analysis. Total blood cell counts were calculated by using 15 µm polystyrene microbeads (Polysciences,

18328-5). The number of CD4+ T lymphocytes (P16 gate, also gated on CD3), CD8+ T lymphocytes (P8 gate, also gated on CD3) and CD19+ MHCII+ B cells (P19 gate) were no different between control and microglia-depleted animals. Similarly, the number of granulocytes (P17 gate, CD11b+, Ly6c+, SSC^{high} cells), CD11b+ $Ly6c^{vhigh}$ monocytes (P9 gate), $CD11b_{low}$ $Ly6c^{vhigh}$ cells (P14 gate) and CD11b+ $Ly6c_{low}$ cells (P23 gate) were no different between control and microglia depleted mice. Data are expressed as mean ± s.e.m. Unpaired t-test, n=4-5. n.s. – not significant.

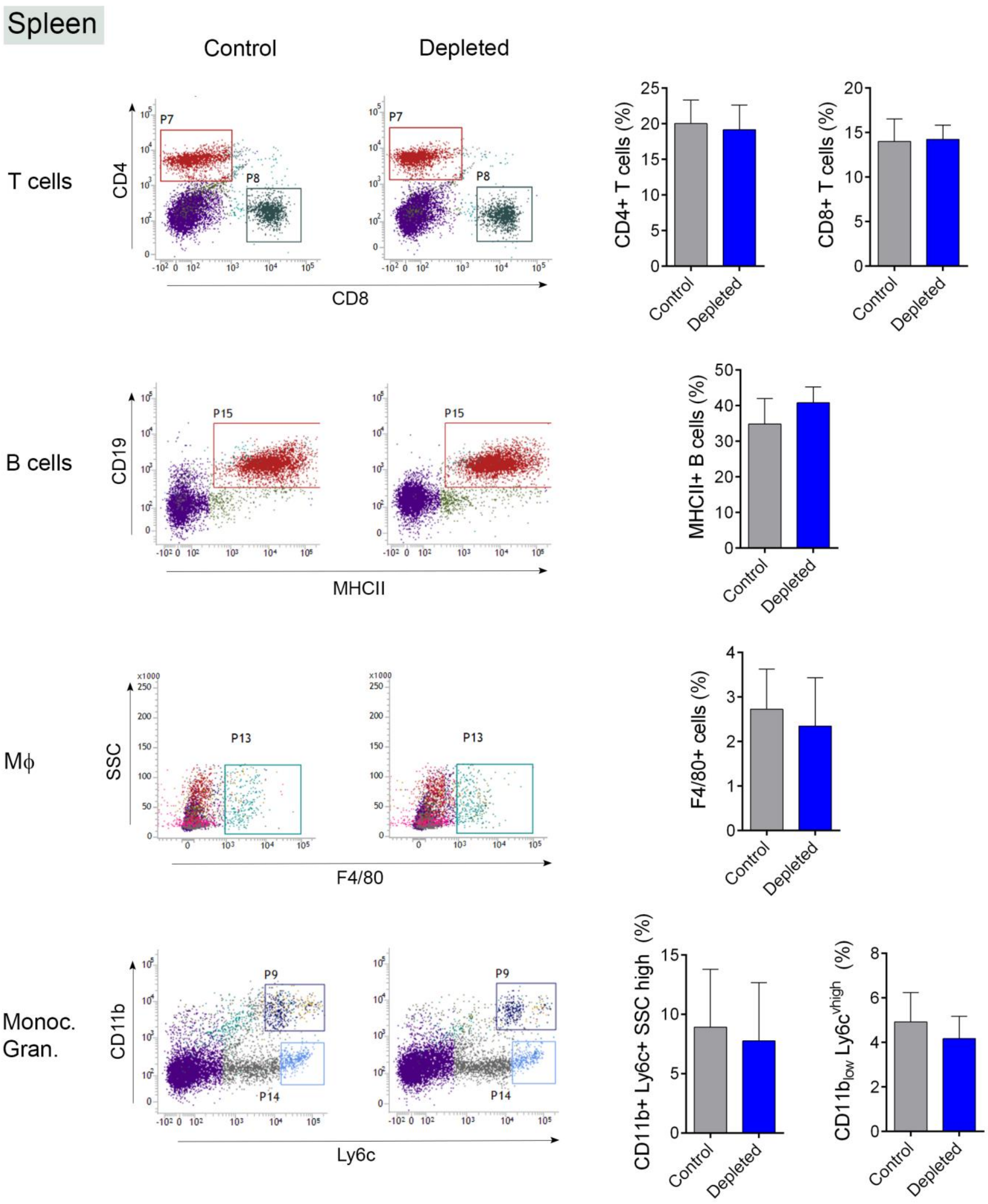

Supplementary Figure 4. **Selective depletion of microglia does not alter main leukocyte populations in the spleen.** C57BL6/J mice were fed a chow diet for 21 days containing the CSF1R antagonist PLX3397 (290 ppm). Spleen cells were isolated and labelled with mixtures of specific antibodies against leukocyte populations followed by flow cytometric analysis. The proportion of CD4+ T lymphocytes (P7 gate, also gated on CD3),

CD8+ T lymphocytes (P8 gate, also gated on CD3) and CD19+ MHCII+ B cells (P15 gate) in the spleen were not different between control and microglia depleted animals. Spleen macrophages (F4/80+ cells), granulocytes (P9 gate, CD11b+, Ly6c+, SSC^{high} cells) and CD11b$_{low}$ Ly6c^{vhigh} monocytes (P9 gate) were not different between control and microglia depleted animals. Data are expressed as mean ± s.e.m., Unpaired t-test, n=4-5.

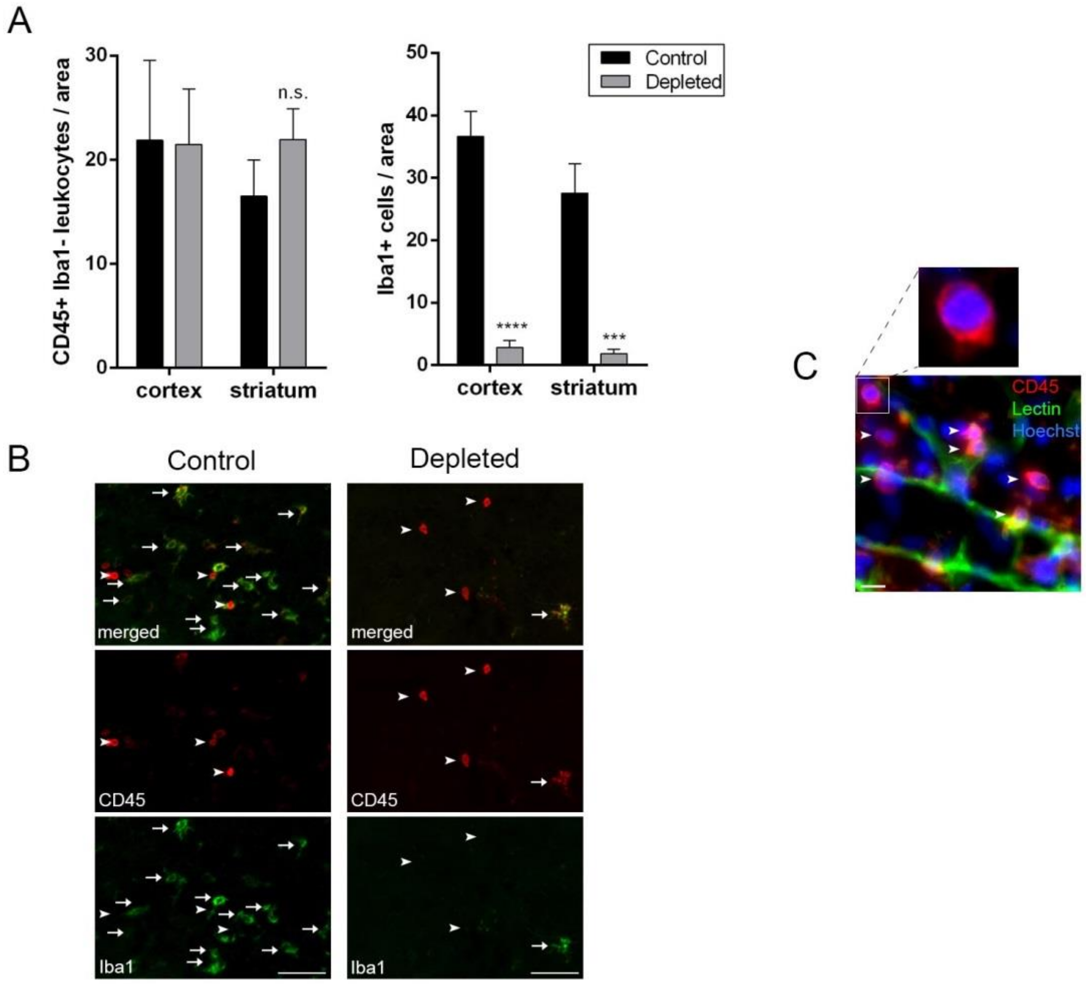

Supplementary Figure 5. Depletion of microglia does not alter leukocyte recruitment into the brain 72h after cerebral ischemia. C57BL6/J mice were fed a chow diet containing the CSF1R antagonist PLX3397 (290 ppm) or control diet for 21 days and were subjected to cerebral ischemia and 72 h reperfusion. **A**. CD45-positive, Iba1-negative leukocytes and Iba1-positive microglia/macrophages were counted in the ipsilateral striatum and the cerebral cortex. **B**. Fluorescent images showing CD45-positive Iba1-negative leukocytes

(arrowheads) and Iba1-positive microglia/macrophages (arrows) in the ipsilateral cerebral cortex. **C**. Image showing CD45-positive leukocytes (arrowheads, red) associated with a blood vessel (tomato lectin, green), cell nuclei were labelled with Hoechst (blue). ***P<0.001, ****P<0.0001, two-way ANOVA followed by Sidak's multiple comparisons test. Scale bars: B, 50 μm; C, 10 μm. Data are expressed as mean ± s.e.m., n=8. A: two-way ANOVA followed by Dunn's multiple comparison. n.s. – not significant.

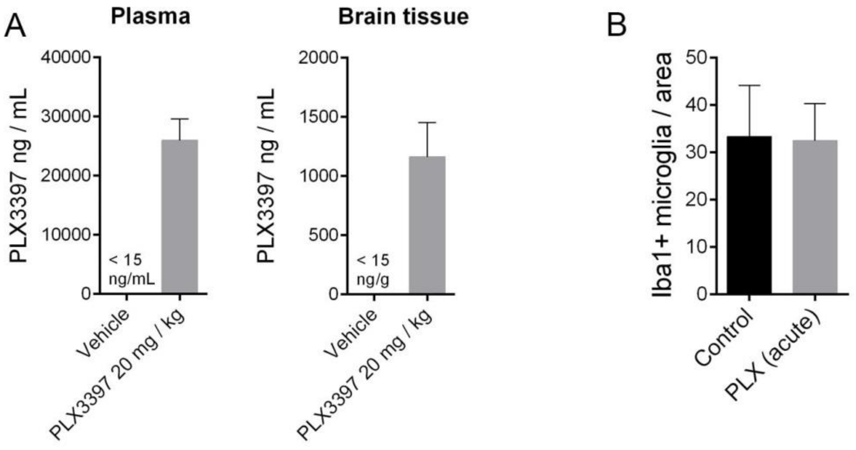

Supplementary Figure 6. **Acute treatment with PLX3397 does not reduce microglial numbers within 24 h. A**. For pharmacokinetic studies, PLX3397 (20 mg/kg) or vehicle (PBS) was administered intraperitoneally to control C57BL6/J mice 2h before sample collection (n=3). PLX3397 levels were measured by extraction into acetonitrile (containing an internal standard) and quantification by liquid chromatography-tandem mass spectrometry (LC/MS/MS). **B**. C57BL6/J mice were subjected to 45 min MCAo and received an intraperitoneal injection of either vehicle or PLX3397 (20 mg/kg) 60 min after occlusion. 24 h after reperfusion mice were sacrificed and Iba1-positive cell numbers determined in the cerebral cortex. Data are expressed as mean ± s.e.m., A: n=3; B: unpaired t test, n=7.

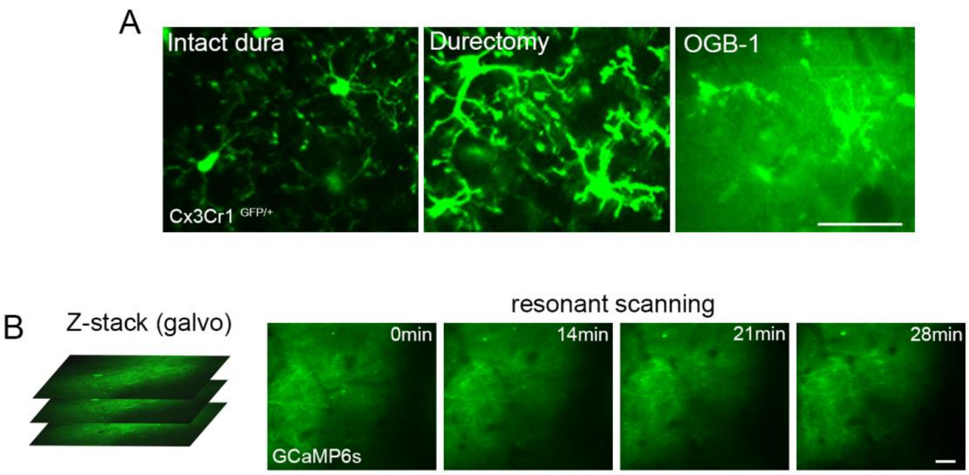

Supplementary Figure 7. Effect of genetically encoded calcium indicator delivery on microglial activation and two-photon imaging in the cerebral cortex 24h after cerebral ischemia. **A**. Injection of genetically encoded calcium indicators into the brain parenchyma was performed distant from the imaging area at a 60° angle, to minimize disturbance to the brain tissue. This protocol does not induce microglial activation at the imaging site, unless durectomy is performed, as assessed in Cx3Cr1$^{GFP/+}$ microglia reporter mice. Injection of the calcium indicator Oregon Green 488 BAPTA-1 (OGB-1) prior to *in vivo* two-photon imaging leads to microglial activation based on withdrawal of cellular processes and enlargement of the cell body. **B**. *In vivo* neuronal calcium imaging 24 h after MCAo in the cerebral cortex reveals very few changes in GCaMP6s signal over time. Scale bars: 50μm.

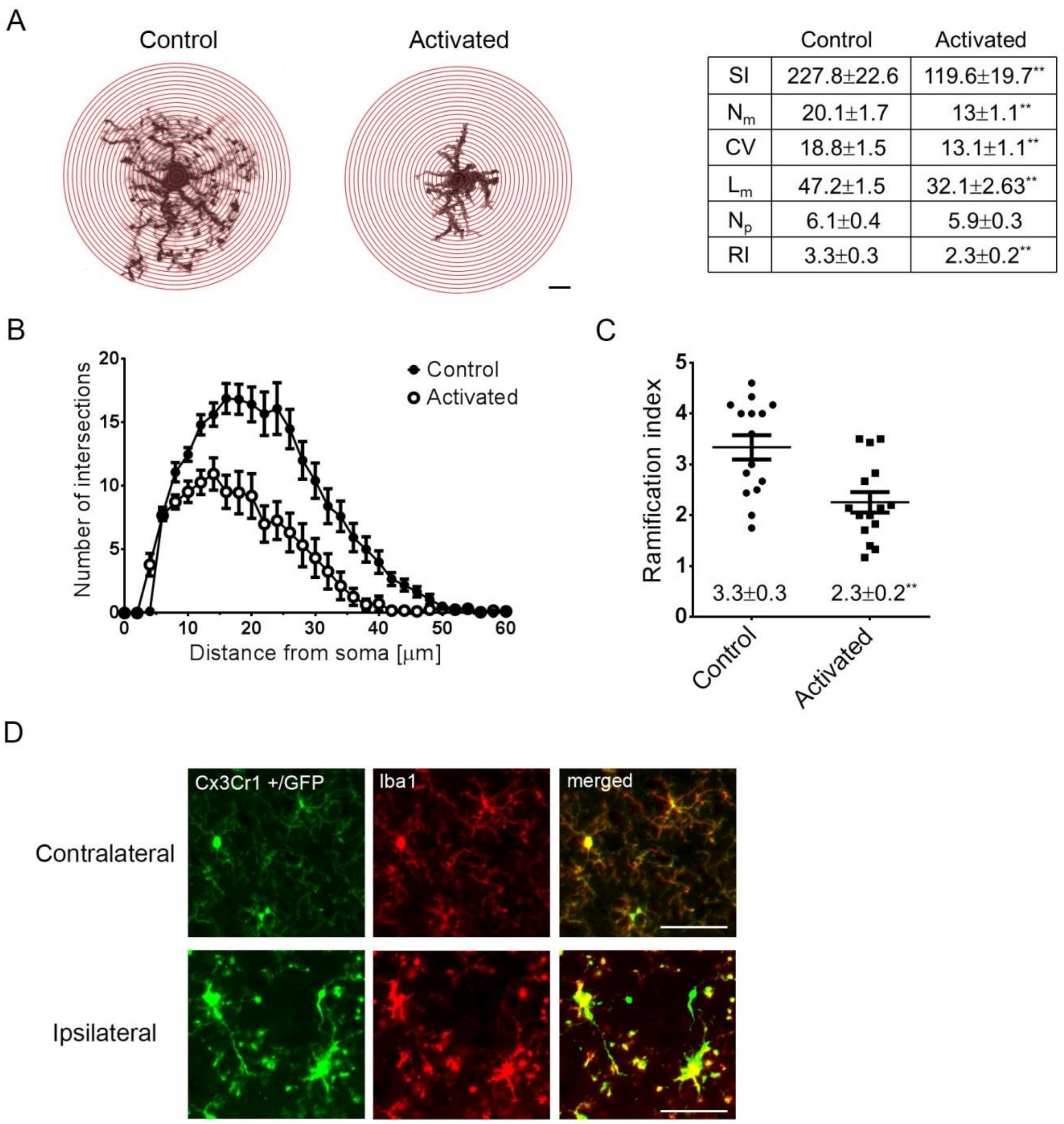

Supplementary Figure 8. **Microglial morphology is altered rapidly in response to cerebral ischemia. A.** Representative diagrams used for classical Sholl analysis showing microglial cells from a control mouse and 1h after cerebral ischemia (activated) in the cerebral cortex. Microglia in Cx3Cr1$^{+/GFP}$ reporter mice were visualized by *in vivo* two-photon imaging. Series of concentric circles were spaced at 2 µm intervals from the center of the soma. The number of process intersections with each circle was counted. SI = Sum of Intersections, the total number of intersections per cell; N_m = process maxium, the maximum number of intersections at radius r; CV = critical value, the radius r [µm] with the maximum number of intersections; N_p = the number of primary branches originating at the cell's soma;

RI = Schoenen Ramification Index, a measure of the branching of the cell calculated by dividing the process maximum by the number of primary branches; L_m = maximum branch length, the maximum radius where an intersection with a process occurs (μm). **B.** Number (mean±s.e.m.) of intersections in control (n=15) and activated (n=15) microglial cells as a function of distance from the soma. **C.** Schoenen Ramification Index, a measure of the branching of the cells was calculated by dividing the maximum process number by the number of primary branches. The data indicate significantly reduced branching in response to cerebral ischemia. (**p=0.0017, unpaired t-test). **D**. Histological analysis in $Cx3Cr1^{+/GFP}$ mice indicates higher Iba1 (red) expression in activated microglial cells 6 h after cerebral ischemia. Scale bars: A, 10 μm; D, 50μm.

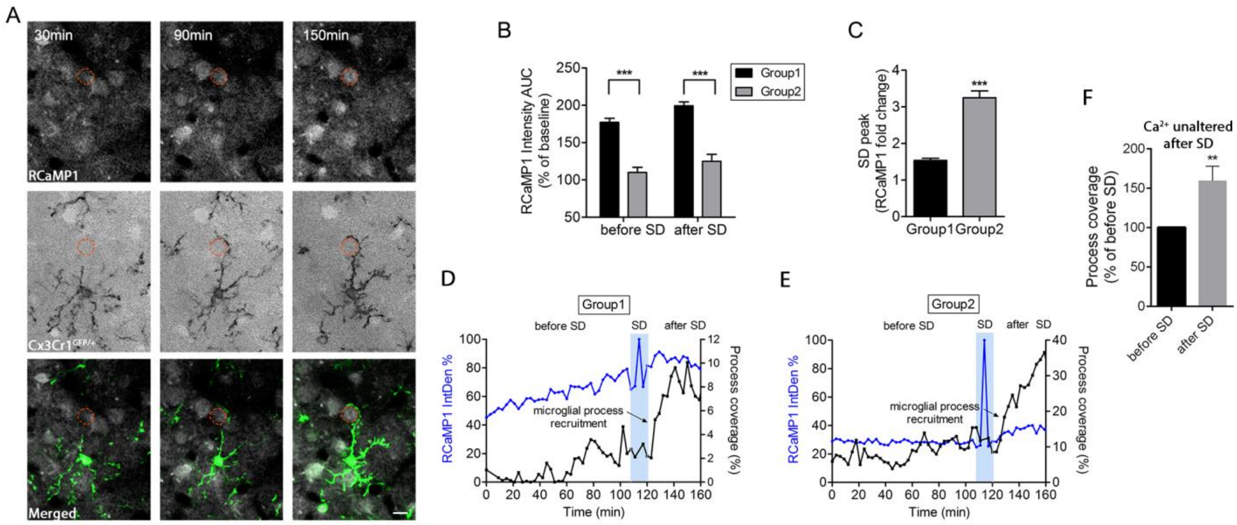

Supplementary Figure 9. Microglial process coverage of neuronal cell bodies follows neuronal calcium changes and SD after cerebral ischemia. A. *In vivo* two-photon imaging was performed every 3 min (Z-stacks recorded with galvo scanning) starting 15 min prior to the onset of cerebral ischemia. Monochromatic images have been thresholded and used for analysis of microglia process coverage (area) of neuronal cell bodies by a custom made concentric circle macro (modified Sholl analysis) that was run using Image J. **B**. After the induction of cerebral ischemia, one population of neurons showed increasing RCaMP1 signal (P<0,0001, two-way ANOVA) over time (termed as ”Group 1” cells, $P_{baseline\ vs\ before\ SD}$<0,0001; $P_{before\ vs\ after\ SD}$<0,05; $P_{baseline\ vs\ after\ SD}$<0,0001 one-way ANOVA) whereas another population displayed minimal RCaMP1 signal increases over baseline (termed as ”Group 2” cells) before and after SD. **C**. Group 2 cells, however, showed a larger RCaMP1 signal increase during SD (unpaired t test, P<0.0001). **D**. Representative graph showing Group 1 cell with increasing RCaMP1 signal and increasing microglial process coverage after SD. **E**. Representative graph showing Group 2 cell with negligible RCaMP1 signal changes and increasing microglial process coverage after SD. **F**. Neurons with no significant RCaMP1 signal change after SD (Group 2 cells) show significantly increased microglial process recruitment in response to SD (unpaired t test, P<0.01). Data are expressed as percentage of baseline (n=33 Group 1 and n=17 Group 2 neurons).

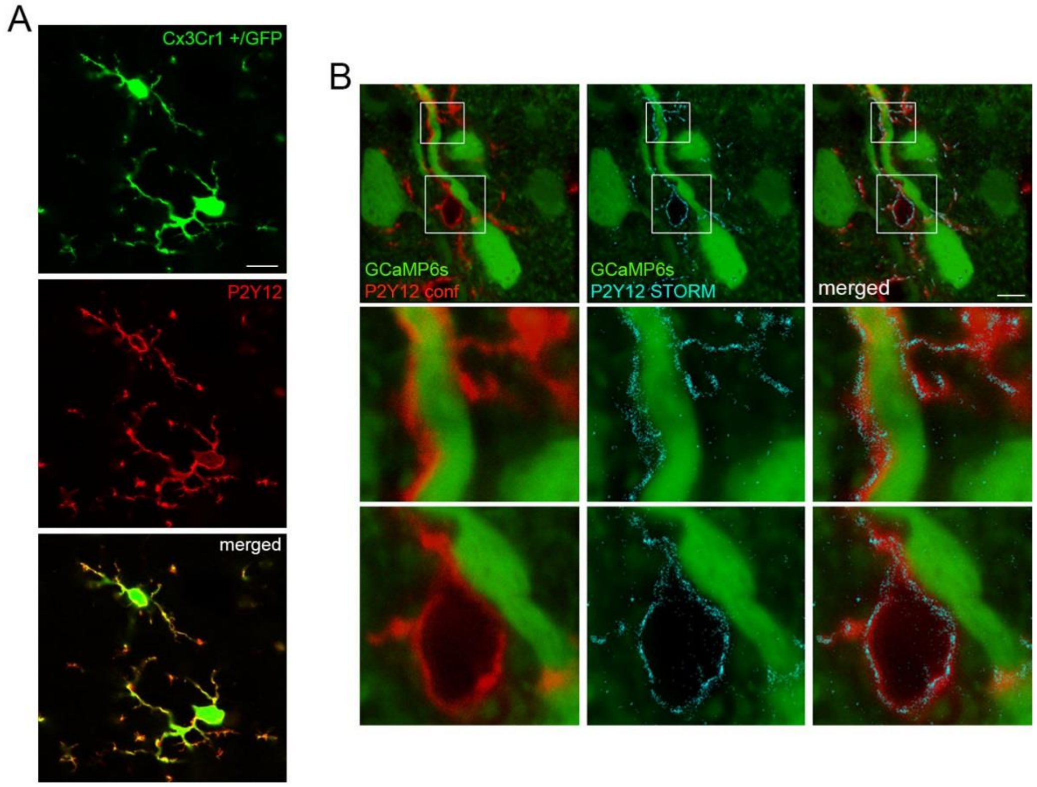

Supplementary Figure 10. Super-resolution microscopy reveals sites of microglia-neuron interactions in the brain. **A**. Confocal images showing a precise colocalisation between GFP signal in Cx3Cr1$^{GFP/+}$ microglia reporter mice and P2Y12 receptors. **B**. STORM super-resolution microscopy identifies clusters of P2Y12 receptors (P2Y12 STORM, cyan) overlapping with confocal images of P2Y12 –positive microglial processes (P2Y12 conf., red). Microglial processes come to close contact with GCaMP6s-positive neurons. Images were captured in the cerebral cortex of a mouse subjected to sham surgery. Note the sparse microglial labelling around the neuronal cell bodies compared to that seen in mice after cerebral ischemia (Fig. 3I, J). Scale bar: A, 10 μm; B, 5 μm.

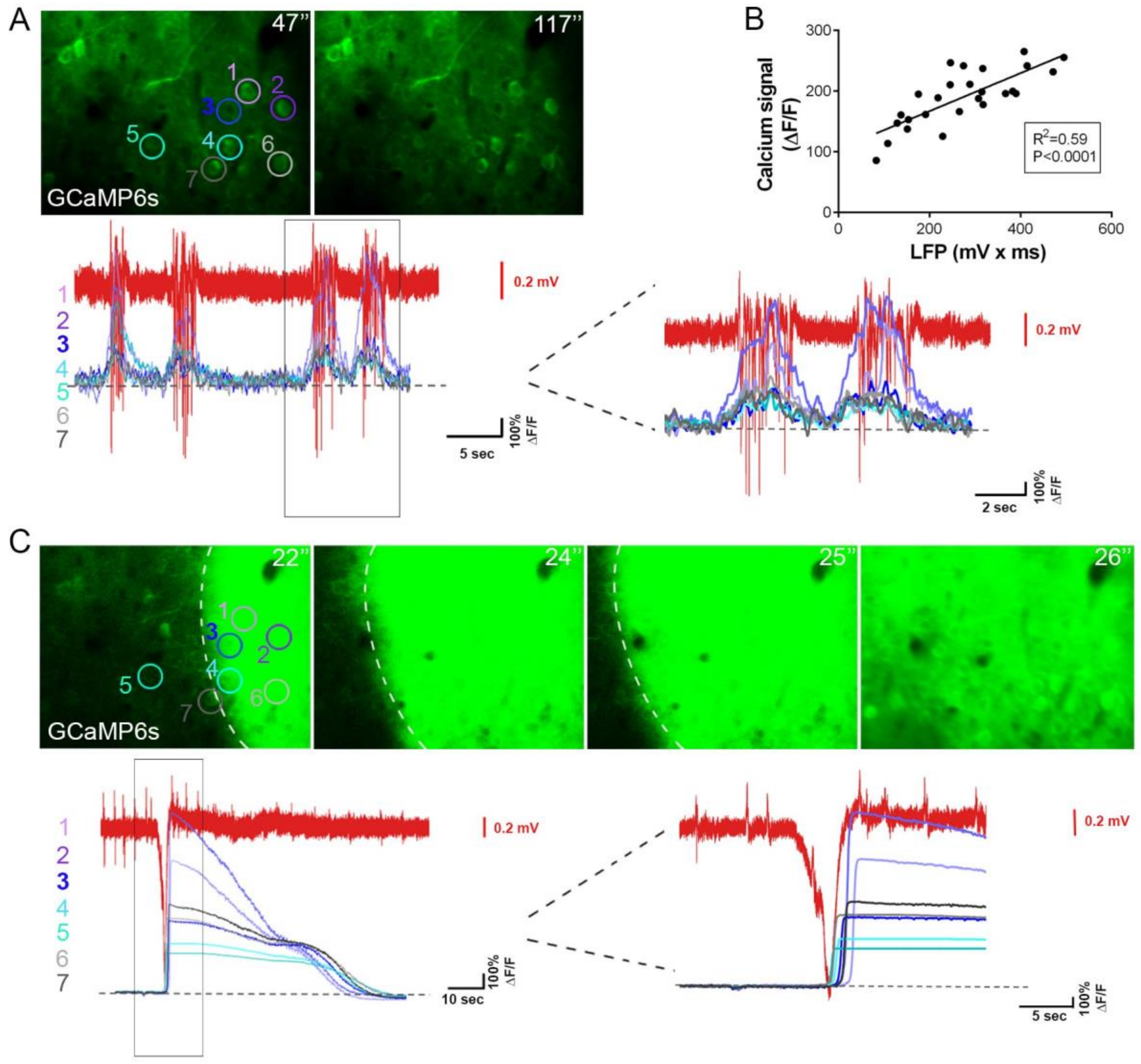

Supplementary Figure 11. Local field potential measurements correlate with GCaMP6s signal changes in the brain *in vivo*. Local field potential (LFP) was recorded with a borosilicate glass electrode (~0.1 MΩ) filled with ACSF. Neuronal calcium responses were recorded with resonant scanning using GCaMP6s labelling. Simultaneous LFP recordings were performed in IC mode at 0 mV holding potential. The distance between the pipette and the imaging area was approximately 100-150 μm. **A**. Spontaneous calcium responses and LFP recorded prior to KCl application. Calcium curves from 7 representative neurons (circled) are shown. **B**. Correlation between LFP (area under the curve, mV x ms) and calcium signals expressed as an average ΔF/F of 11 neurons (area under the curve) is shown for 26 spontaneous burst events. **C**.100 mM KCl was applied topically to the surface of the brain and GCaMP6s signal recorded in parallel with LFP measurements.

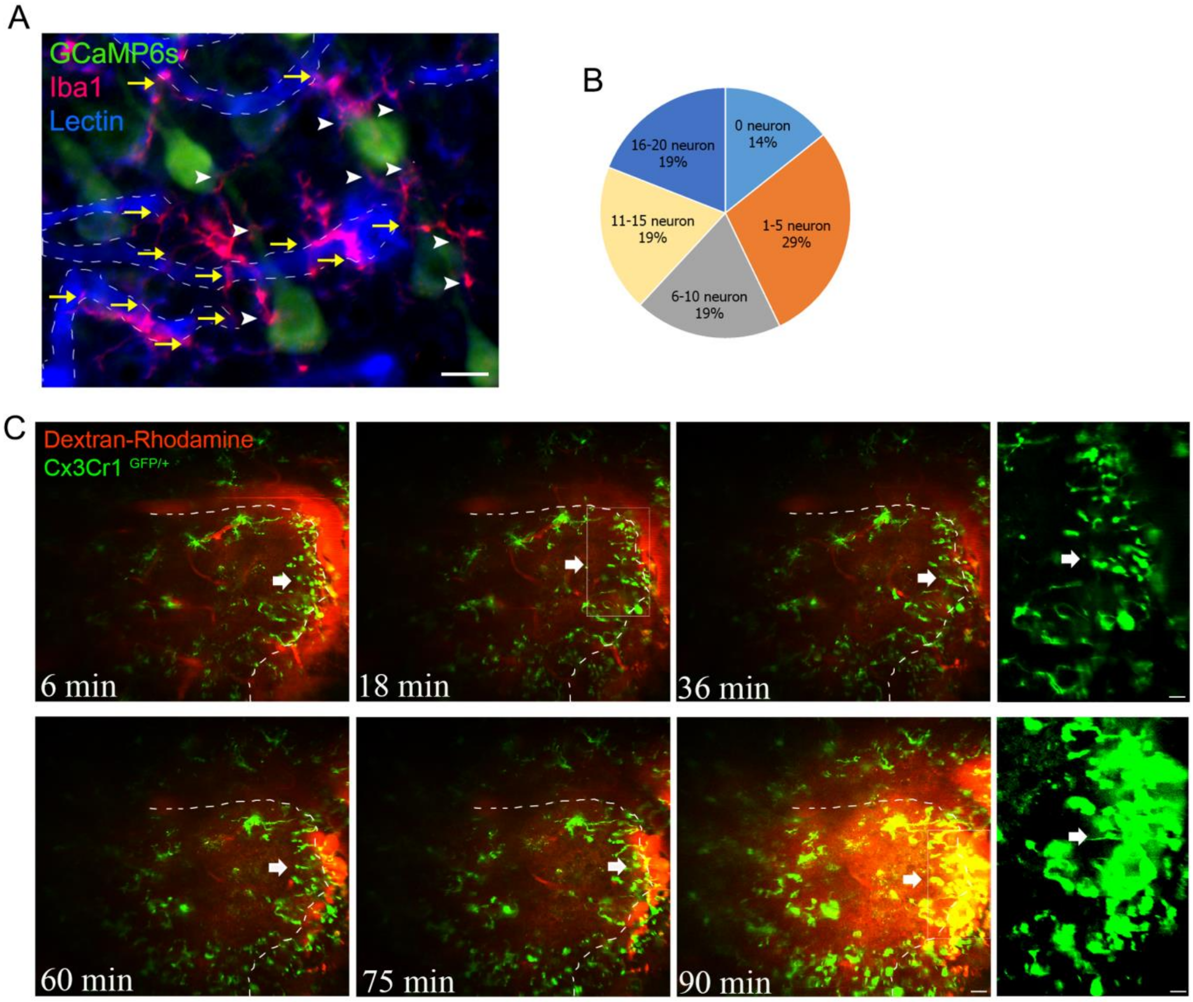

Supplementary Figure 12. **Microglia rapidly react to changes in blood brain barrier permeability after cerebral ischemia. A**. Immunofluorescent characterization of microglia reveal an association between microglial processes (Iba1, red) and GCaMP6s expressing neurons (green, arrowheads) as well as between microglial processes and capillaries (blue, arrows). **B**. Pie chart showing percentage of microglia contacting different numbers of neurons based on *in vivo* two-photon imaging data (see Figure 2) and histological assessment. **C**. *In vivo* two-photon imaging in Cx3Cr1$^{GFP/+}$ microglia reporter mice showing that micoglia isolate sites of BBB injury (red, Dextran-Rhodamine, 70,000 MW) and transform to an amoeboid shape in close vicinity of injured blood vessels. Times indicate minutes after the induction of cerebral ischemia. Scale bars: A, 10 μm; C, 20 μm.

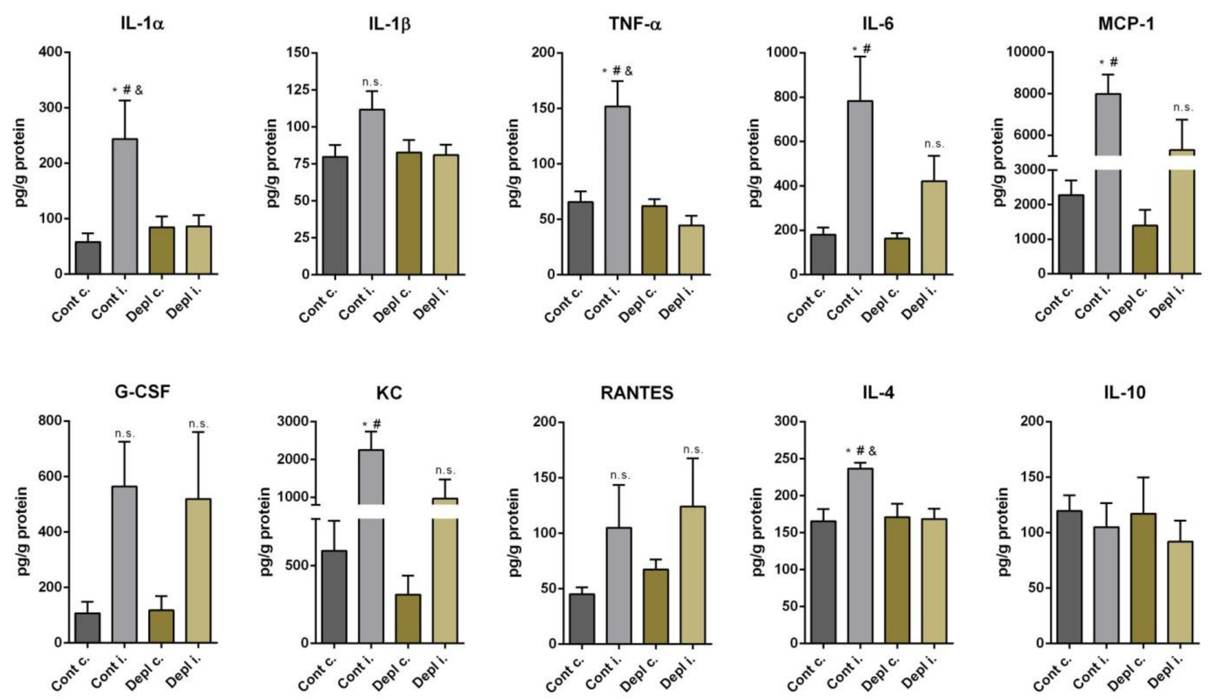

Supplementary Figure 13. Changes in cytokine production after cerebral ischemia in the brain. Cytokine levels in homogenates of the ipsilateral (i) and contralateral (c) hemispheres were measured by cytometric bead array in control (cont) and microglia depleted (depl) mice, 8h after the onset of cerebral ischemia. Mice were transcardially perfused with saline prior to the collection of brain samples. IFNγ was not detectable in the brain (detection limit of the assay was 5 pg/ml), hence values are not shown. ANOVA followed by Tukey's post hoc test, $^{*}P<0.05$ vs Cont c., $^{\#}P<0.05$ vs Depl c., $^{\&}P<0.05$ vs Depl i., Data are expressed as mean ± s.e.m., n=5 mice per group. n.s. – not significant.
